# A Low-Potential π‑Extended Viologen Electron Donor Results in Increased H_2_ Production by *Cp*I [FeFe]-Hydrogenase

**DOI:** 10.1021/acsorginorgau.5c00106

**Published:** 2026-01-20

**Authors:** Nils Ostermann, Sophie Webb, Andrea Do Nascimento Henriques, Ross D. Milton

**Affiliations:** † National Centre of Competence in Research (NCCR) Catalysis, 27212 University of Geneva, 1211 Geneva 4, Switzerland; ‡ Department of Inorganic and Analytical Chemistry, Sciences II, 27212 University of Geneva, Quai Ernest-Ansermet 30, 1211 Geneva 4, Switzerland

**Keywords:** hydrogen evolution reaction, [FeFe]-hydrogenase, viologen, CpI, CrHydA1, mediated electron transfer

## Abstract

[FeFe]-hydrogenases are known for their exceptional dihydrogen production activity, which opens a pathway to green hydrogen formation. To achieve the high turnover numbers found in natural environments, researchers aim to mimic biological electron donors using artificial electron delivery systems. In this sense, the use of an externally applied electrochemical potential is of particular interest, for which redox mediators often have to be included to effectively shuttle electrons between the electrode and the enzyme. The class of viologens, with methylviologen being the most prominent example, has been shown to perform efficient electron transfer. However, the reduced forms of such viologens are restricted in terms of stability and accessibility of the more reduced, low-potential species. Herein, we report the electrochemical and spectroscopic characterization of a viologen-based derivative, which is capable of storing two electrons at a low potential of −0.75 V *vs*. SHE. In combination with the [FeFe]-hydrogenase *Cp*I, an activity of up to 10,480 μmol_H_2__ mg_*Cp*I_^–1^ min^–1^ (molar activity of 11,179 μmol_H_2__ μmol_*Cp*I_^–1^ s^–1^) could be achieved, which is 10 times greater than the commonly used dithionite-driven methylviologen assay under the same conditions.

## Graphical abstract

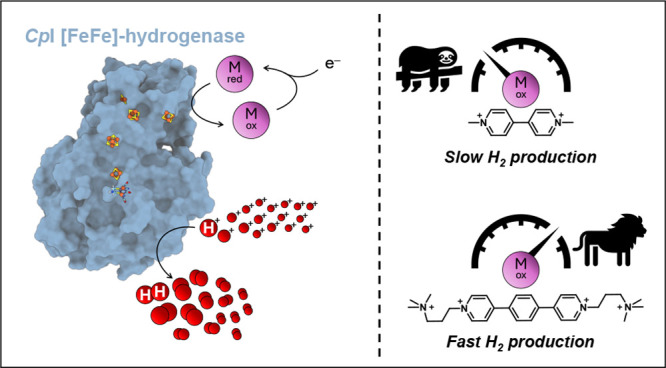

## Introduction

[NiFe]- and [FeFe]-hydrogenases, named after their metal cofactors, catalyze the reversible interconversion of protons (H^+^) and dihydrogen (H_2_), according to eq 1.^1^

$$2H^{+}+2e^{-}⇌[H^{+}+H^{-}]⇌H_{2}$$ 1

For the hydrogen evolution reaction (HER), *in vivo* electrons are delivered to hydrogenases by reduced electron-transferring proteins, *i.e.*, ferredoxin (Fd), flavodoxin (Fld), and cytochrome *c*_3_ (cyt *c*_3_), or coenzymes, specifically nicotinamide adenine dinucleotide (NADH, Table 1).^2^ *In vitro*, the reducing equivalents are commonly supplied by sacrificial reducing agents, such as dithionite (S_2_O_4_^2–^, DT; Table 1). However, the electron transfer (ET) rate constant (*k*_ET_) with DT is low, and an electron mediator (*e.g.*, methylviologen (*N,N*′**-dimethyl-4,4*′*-bipyridinium, MV), *vide infra*) is often included to shuttle electrons from DT to hydrogenases, thereby increasing *k*_ET_ and the specific activity of the enzyme.^3^ A desirable, alternative approach for an electron donor is to apply a reductive electrochemical potential.^4^ However, wiring an enzyme to an electrode surface and delivering reducing equivalents by direct electron transfer (DET) from the electrode with a significant heterogeneous *k*_ET_ can be challenging due to a number of issues.^5^ To efficiently transfer electrons, DET requires not only a surface-exposed redox-active subunit, typically an FeS cluster, but also an optimal orientation of the hydrogenase enzyme on the electrode surface. With this, the distance between the FeS cluster and the electrode surface can be minimized (Figure 1), thereby increasing *k*_ET_.^6^ Although there has been remarkable progress in DET approaches, as is the case for [FeFe]-hydrogenases,^7^ the use of a synthetic redox mediator is still commonly employed to study enzyme activities in solution and on electrode surfaces. During mediated electron transfer (MET, Figure 1), a small diffusive redox active molecule shuttles electrons from the electrode to the enzyme, and the resulting ET process is largely independent of the enzyme orientation at the electrode surface.^5^

**Figure 1:**
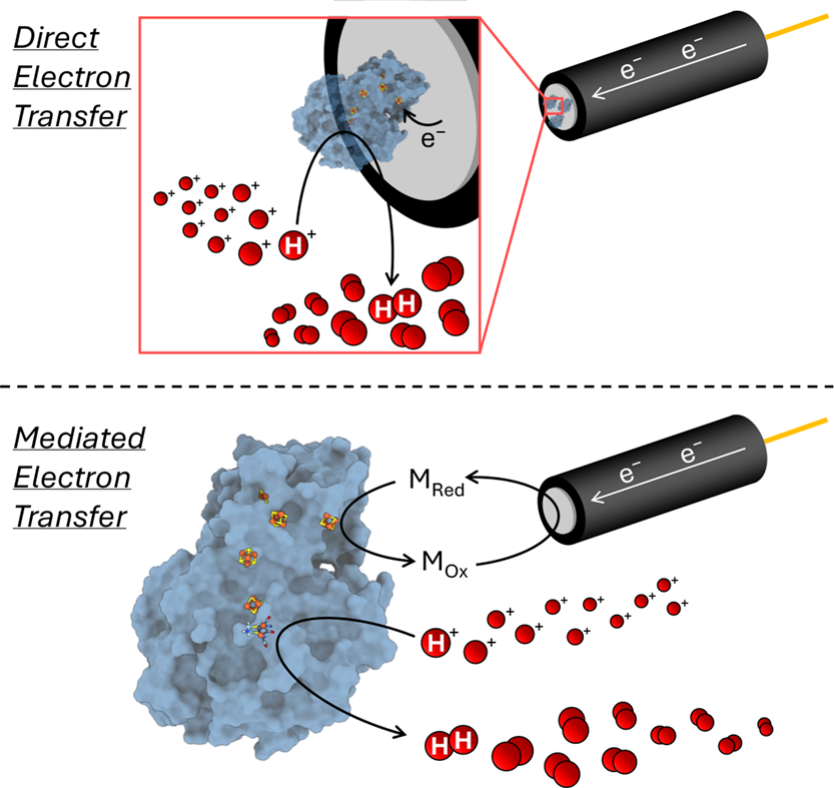
Schematic representation of electron transfer to an enzyme (here, [FeFe]-hydrogenase *Cp*I, PDB: 6N59). Top: direct electron transfer (DET) with the enzyme immobilized on the electrode surface; bottom: mediated electron transfer (MET) with the enzyme being in solution together with a redox mediator M_red_/M_ox_.

**Table 1:** Biological Standard Reduction Potentials (*E* ^0^') of Selected Biologically Relevant Compounds and Artificial Electron Donors Commonly Employed for [FeFe]-Hydrogenases

electron donor	*E* ^0^' [V *vs.* SHE]	reference
ferredoxin	<−0.40	^12^
flavodoxin	∼−0.45	^13^
cytochrome *c*_3_	–0.36 to −0.13	^14^
NADH	–0.32	^12^
dithionite	∼−0.66	^15^
methylviologen	–0.44	^16^ and this work
triquat	–0.54	^17^ and this work

Such mediators need to fulfill a list of criteria to be suitable for efficient and sustainable ET between the electrode and the enzyme: (i) having a reduction potential (*E* ^0^') that is ideally more negative than the electron-accepting subunit of the enzyme (for a reductive reaction), (ii) having sufficient solubility and stability in the enzyme media, where high performance catalysis occurs, (iii) having a suitable charge and size to effectively shuttle electrons through the protein network, and (iv) being a fully reversible redox species.^5^ In this regard, bipyridine-based compounds like triquat (*N,N′*-trimethylene-2,2*′*-bipyridinium, TQ) or viologen derivatives have been shown to be potent redox mediators, with MV being the most prominent and studied candidate (Table 1).^8–10^ However, the reduced forms of MV^2+^ also exhibit certain chemical disadvantages, *i.e.*, the second, low-potential reduction is not practical in aqueous media due to the insolubility of the neutral MV^0^ species, and the singly reduced radical cation MV^+•^ tends to react with itself in an unfavorable dimerization, which ultimately disproportionates into oxidized MV^2+^ and insoluble MV^0^ (Figure 2).^11^ (Note: "diquat" (DQ) has also been used in the literature to describe the class of molecules named as TQ above, with both dimethylene and trimethylene bridges used for the di-quaternization of the *N* atoms in the 1,1'- positions. Methylviologen, a.k.a. paraquat, is also di-quaternized ("diquat") but does not follow this naming convention. For these reasons, TQ has been used for these molecules containing the trimethylene bridge between the two quaternized *N* atoms in the 1,1'- positions.)

**Figure 2:**
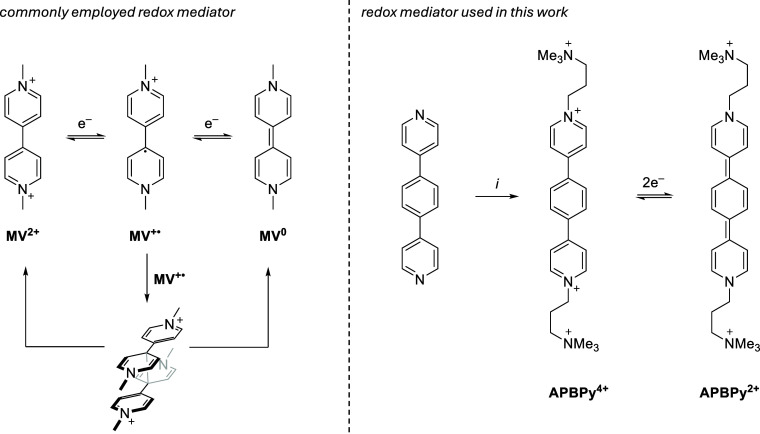
Left: reaction scheme upon two consecutive one-electron reductions of MV^2+^. The arrows from the dimer to MV^2+^ and MV^0^ indicate its disproportionation. Right: reaction scheme of the viologen mediator used in this work and its two-electron reduction to the dication. *i*) (3-bromopropyl)­trimethylammonium bromide in DMF, followed by anion exchange. Counteranions have been omitted for clarity.

Herein, we report the characterization of a low-potential viologen derivative that benefits from decoupled reductions due to spatial separation of the two pyridine subunits. Bearing two charged side groups not only allows the doubly reduced viologen to be water-soluble but also prevents dimerization of the radical cation one-electron-reduced species. Here, we examine the use of this viologen species as an electron donor to [FeFe]-hydrogenases.

## Results and Discussion

### Electrochemical and Spectroscopical Characterization

The electron mediator, 1,1′-bis­(3-(trimethylammonium)­propyl)-4,4′-(1,4-phenylene)-bipyridinium tetrachloride ([**APBPy**]­Cl_4_), was prepared according to a modified literature procedure (Figure 2, see the Experimental Section for further details).^18^ Initially, cyclic voltammetry (CV) was used to determine the *E* ^0^' of [**APBPy**]^4+^. The CV trace shows a single, diffusion-controlled reversible reduction wave at *E*_1/2_ = −0.75 V *vs.* SHE, which is cathodically shifted by 310 and 210 mV compared to the first reduction of the related MV^2+^ and TQ^2+^ species, respectively (Figure 3, Figure S6).[Fn fn1] Assuming a similar diffusion coefficient of [**APBPy**]^4+^ to MV^2+^, and according to the Randles–Ševčík equation for diffusion-controlled redox processes of freely diffusing species (eq 2),^19^ the relative increase of the peak currents in the CV trace of [**APBPy**]^4+^ at the same concentration as MV^2+^ and TQ^2+^ indicates a multiple electron process for the reduction wave at *E*_1/2_ of [**APBPy**]^4+^.

$$i_{p}=0.446n{\mathrm{FAC}}_{0}\sqrt{\frac{nF\nu D_{0}}{RT}}$$ 2

**Figure 3:**
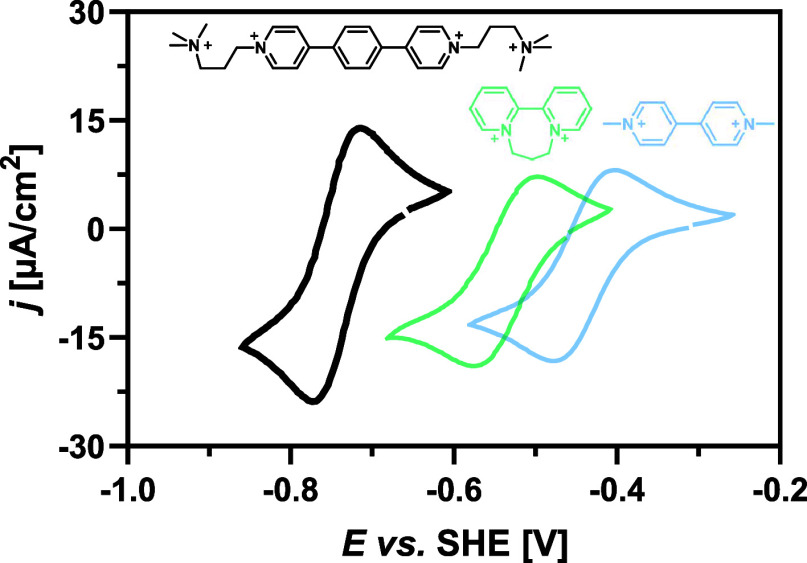
CV traces of MV^2+^ (faint blue), TQ^2+^ (faint green), and [**APBPy**]^4+^ (black) in aqueous NaCl (0.1 M), *c*_0_ = 0.5 mM, *v* = 2.5 mV s^–1^, glassy carbon (GC) working electrode, Pt counter electrode, SCE reference electrode (converted to SHE by applying the conversion factor of +0.241 V), plotted in IUPAC convention.

The diffusion coefficient (*D*_0_) was determined by diffusion-ordered NMR spectroscopy (DOSY) to be ∼2.90 × 10^–6^ cm^2^ s^–1^ (Figure S4). From scan-rate-dependent CV measurements (Figure S6) and the linear plot of the peak current (*i*_p_) *vs*. the square root of the scan rate (ν), the number of transferred electrons (*n*) could be determined, using eq 2, to be 1.52, in accordance with a more complex multielectron process. Further analysis revealed a peak-to-peak separation of 62 mV (*cf*. 67 mV for MV^2+^, theoretically 57 mV for a 1e^–^, and 28.5 mV for a 2e^–^ process, respectively^19^), likely indicative of two consecutive 1e^–^ reductions at a very similar potential rather than one single 2e^–^ reduction and therefore a weak-yet-existent coupling of the two pyridinium subunits, which is in agreement with selected literature reports.^20–22^ Attempts to electrochemically discern the two reductions were unsuccessful, indicating that both reductions are indeed very close in potential. Furthermore, it is hypothesized that the extended aromatic system undergoes π-stacking to form dimeric intermediates that might complicate their electrochemical interpretation.^21^ In earlier NMR and EPR studies, upon galvanostatic cycling of a cell containing [**APBPy**]^4+^, the radical trication ([**APBPy**]^3+•^) en route to the doubly reduced dication ([**APBPy**]^2+^) could be observed spectroscopically.^21^ We utilized UV/Vis spectroscopy to further characterize the different oxidation states of the viologen derivative. As expected, fully oxidized [**APBPy**]^4+^ does not present a significant feature in the visible absorption region, merely a high-energy π–π* transition in the UV range of around 350 nm (Figure 4). [**APBPy**]^2+^, which can be prepared *via* bulk electrolysis, shows a strongly absorbing transition from the highest occupied molecular orbital (HOMO) to the lowest unoccupied molecular orbital (LUMO) with an absorption maximum at 544 nm (*ϵ*_λmax_ = 68,182 M^–1^ cm^–1^; Figure S11). Calculations on the unsubstituted H*N*-pyridine congener are in agreement with the obtained spectra and suggest a closed-shell quinoid rather than a diradical ground state structure, which is also likely for [**APBPy**]^2+^.^23^ This assumption is supported by the disappearance of the π–π* transition around 350 nm, indicating the loss of aromaticity, as expected for a quinoid structure. Upon preparation of deep purple [**APBPy**]^2+^, we could observe a yellow/brown transient species with a distinct spectroscopic pattern (Figure S5), which we have tentatively assigned to the radical trication [**APBPy**]^3+•^. [**APBPy**]^3+•^ shows two absorption bands with maxima at 468 and 510 nm, respectively (Figure 4). [**APBPy**]^3+•^ gained a singly occupied molecular orbital (SOMO), and the absorptions can be assigned to SOMO–LUMO and HOMO–SOMO transitions, respectively. This is in agreement with the radical cation of the *N*-pyridine-methyl-substituted congener, in which the bands were assigned accordingly.^24^ Notably, the recorded spectrum could only be observed at a low concentration of [**APBPy**]^3+•^, as upon prolonged reduction [**APBPy**]^2+^ is formed, whose strong absorbance dominates the UV/Vis spectrum. Consequently, attempts to cleanly isolate [**APBPy**]^3+•^ were unsuccessful as we always observed mixtures between [**APBPy**]^3+•^ and [**APBPy**]^2+^, supporting the assumption that the two reduction events are very close in potential. Accordingly, the addition of 1 equiv of [**APBPy**]^4+^ to a solution of [**APBPy**]^2+^, and *vice versa* (adding 1 equiv of [**APBPy**]^2+^ to a solution [**APBPy**]^4+^), did not result in a clean formation of [**APBPy**]^3+•^ but instead a mixture of [**APBPy**]^3+•^ and [**APBPy**]^2+^ (Figure S12).

**Figure 4:**
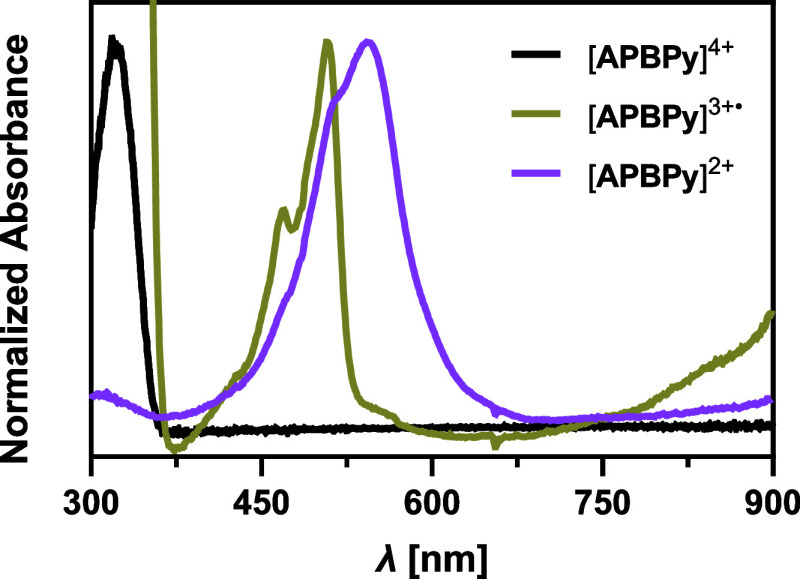
UV/Vis absorption spectra of [**APBPy**]^4+^, [**APBPy**]^3+•^, and [**APBPy**]^2+^ in H_2_O.

Two-electron-reduced [**APBPy**]^2+^ can be prepared by bulk electrolysis in unbuffered aqueous solution using a 0.5 M NaCl supporting electrolyte and stored over several days under an anoxic environment (Figure S5). To investigate the applicability of highly reducing [**APBPy**]^2+^ for enzymatic catalysis, the wider stability was studied at different pH values and in different biological buffer systems. Bulk electrolytically prepared [**APBPy**]^2+^ was added to the respective buffers (at a ratio of 1:1000, which can be readily translated to enzymatic activity measurements), and the optical absorbance at 510 and 544 nm was followed over time. There was no observable pH-dependence in TRIS,[Fn fn2] as indicated by almost no decay in the absorption intensity at both wavelengths (Figure S13). As every tested pH was tolerated, we performed subsequent measurements at pH 8, since the enzyme studied herein ([FeFe]-hydrogenase *Cp*I, *vide infra*) shows the highest activity at this pH.^25^ To further investigate the stability of [**APBPy**]^2+^ in different buffers, other commonly employed biological buffers were examined (MOPS, HEPES, TRIS, TAPS, phosphate buffer, each at 0.01 M and pH 8).[Fn fn2] No clear accelerated decomposition kinetics could be observed (Figure S14), demonstrating the versatility of [**APBPy**]^2+^ as an electron donor. All spectrophotometric measurements show a rather small linear decrease in both absorbances over time, which can be attributed to the oxidation of [**APBPy**]^2+^ by trace impurities of oxygen, highlighting its high reactivity toward oxygen. However, this exceptionally high reactivity can also be beneficial as it might protect oxygen-sensitive enzymes from incoming oxygen. Similar protection strategies have been demonstrated for the hydrogen oxidation reaction by oxygen-sensitive hydrogenases embedded within a viologen-modified polymer matrix by the group of Plumeré.^26,27^

### Electron Transfer to [FeFe]-Hydrogenase(s)

In order to test the compatibility of the electron donor/mediator with metalloenzymes, [FeFe]-hydrogenases were chosen due to their high turnover frequencies (TOFs) for H_2_ evolution of up to 10,000 s^–1^ (the reader is directed to a summary of specific activities for H_2_ evolution^9^).[Fn fn3] The greatest *in vitro* H_2_-evolution activity was observed for the [FeFe]-hydrogenase *Dd*HydAB from *Desulfovibrio desulfuricans* with an activity of 8,200 μmol_H_2__ mg_*Dd*HydAB_^–1^ min^–1^ (corresponding to a specific molar activity of approximately 8,200 μmol_H_2__ μmol_*Dd*HydAB_^–1^ s^–1^, assuming a molecular mass of 60 kDa), using DT and MV as the electron donor and electron mediator, respectively.^29^

[FeFe]-hydrogenase *Cp*I from *Clostridium pasteurianum* is widely studied due to, in part, its ease of heterologous production in *Escherichia coli* (*E. coli*) and its high activity for H^+^ reduction to H_2_, with maximum specific *in vitro* activities of up to 5,500 μmol_H_2__ mg_*Cp*I_^–1^ min^–1^ (or 5,867 μmol_H_2__ μmol_*Cp*I_^–1^ s^–1^, assuming a molecular mass of 64 kDa).^30^ In our hands, activity assays under similar conditions with *Cp*I, heterologously produced here in *E. coli* (Figure S1), yielded a specific activity of 1,968 ± 308 μmol_H_2__ mg_*Cp*I_^–1^ min^–1^ under the following conditions: 400 ng mL^–1^ enzyme, 0.1 M DT (electron donor), 0.01 M MV (electron mediator), 0.1 M TRIS, pH 7, and incubation for 20 min at 37 °C (Figure S16) , which is in accordance with reported activities of *Cp*I heterologously produced in *E. coli*.^31^

To evaluate the use of [**APBPy**]^2+^ as the sole electron source (as opposed to DT as the electron donor and MV as the electron mediator), a bulk electrolytically prepared sample was added to the buffered enzyme (0.02 M TRIS at pH 8 was used to ensure the maximum activity of *Cp*I, *vide supra*). However, when using 400 ng mL^–1^ enzyme alongside 5 mM [**APBPy**]^2+^, rapid decolorization of [**APBPy**]^2+^ was observed (<2 min). Hence, it was concluded that the standard quantity of the enzyme was not conducive to an accurate determination of [**APBPy**]^2+^-driven *Cp*I-catalyzed H_2_ production. For better visualization, a representative example for H_2_ production by *Cp*I (buffered in 0.02 M TRIS, pH 8) was recorded *via* real-time mass spectrometry (RT-MS), as shown in Figure S17. The resulting profiles clearly demonstrate the comparatively accelerated kinetics of [**APBPy**]^2+^ oxidation, even at room temperature, *vs*. MV^+•^ oxidation at 37 °C by *Cp*I (note that in the RT-MS profile, using [**APBPy**]^2+^, the amount of enzyme was already reduced to 40 ng mL^–1^ in order to sufficiently resolve the onset of H_2_ production by *Cp*I, *vide infra*).

In order to observe relatively linear H_2_ production on the minute time-scale using 5 mM of the electron donor (thereby improving the accuracy of specific activity determination), the quantity of *Cp*I was lowered and the resulting activity was initially monitored *via* RT-MS at room temperature. The above-mentioned 10-fold decrease in the quantity of *Cp*I to 40 ng mL^–1^ per assay still resulted in rapid mediator decolorization (<5 min, Figure S17). Further reduction of the *Cp*I loading to 10 ng mL^–1^ per assay permitted more accurate specific activity determination at room temperature over 5 min (Figure S18) and was therefore deemed suitable for subsequent specific activity determination by GC-TCD (gas chromatography-thermal conductivity detector) assays. Employing these optimized conditions (*i.e.*, 10 ng mL^–1^ of enzyme, 5 mM [**APBPy**]^2+^, 0.02 M TRIS, pH 8, and incubation for 5 min at 37 °C), a specific activity for H_2_ production by *Cp*I of up to 10,480 ± 1,607 μmol_H_2__ mg_*Cp*I_^–1^ min^–1^ (11,179 μmol_H_2__ μmol_*Cp*I_^–1^ s^–1^) was observed (Figure 5), demonstrating that [**APBPy**]^2+^ can be used as a potent electron donor to [FeFe]-hydrogenases. For comparison, the activity of *Cp*I under comparable conditions with an equivalent quantity of enzyme using DT/MV with an equimolar amount of electrons, *i.e*., 10 mM DT and 1 mM MV, results in a specific activity of 1,116 ± 236 μmol_H_2__ mg_*Cp*I_^–1^ min^–1^ (Figure 5). The slight decrease in *Cp*I activity with DT/MV under these optimized conditions compared to the standard protocol mentioned in the beginning of this chapter (1,968 μmol_H_2__ mg_*Cp*I_^–1^ min^–1^) can be rationalized by a decrease in saturation of *Cp*I by the reduced MV^+•^ electron mediator, whose ratio is expected to be sensitive due to the similarity in *E* ^0^' between *Cp*I’s distal FeS cluster (<−0.45 V, thought to be the cluster responsible for ET with redox partners) and MV (−0.44 V).^32^ In order to directly compare electrochemically reduced [**APBPy**]^2+^ as an electron donor with the parent MV system, activity measurements with electrochemically reduced MV^+•^ were also performed. Under a similar total electron concentration and identical reaction conditions, *Cp*I shows an activity of 650 ± 294 μmol_H_2__ mg_*Cp*I_^–1^ min^–1^ (Figure 5), still being significantly lower than [**APBPy**]^2+^, showcasing the improved performance of [**APBPy**]^2+^ as an electron donor. Importantly, no H_2_ production was observed using either H-cluster-deficient apo-*Cp*I or using [**APBPy**]^2+^ alone (Figure 5). We also evaluated whether [**APBPy**]^2+^ can be generated *in situ* by mediated electrocatalysis (MET), whereby a carbon electrode first reduces [**APBPy**]^4+^ that in turn delivers electrons to *Cp*I for catalysis (Figure S10). Unfortunately, immediate adsorption of *Cp*I to the glassy carbon electrode surface (from the bulk solution) resulted in DET, and the resulting electrocatalytic response overshadows a detailed interpretation of MET by [**APBPy**]^2+^.

**Figure 5:**
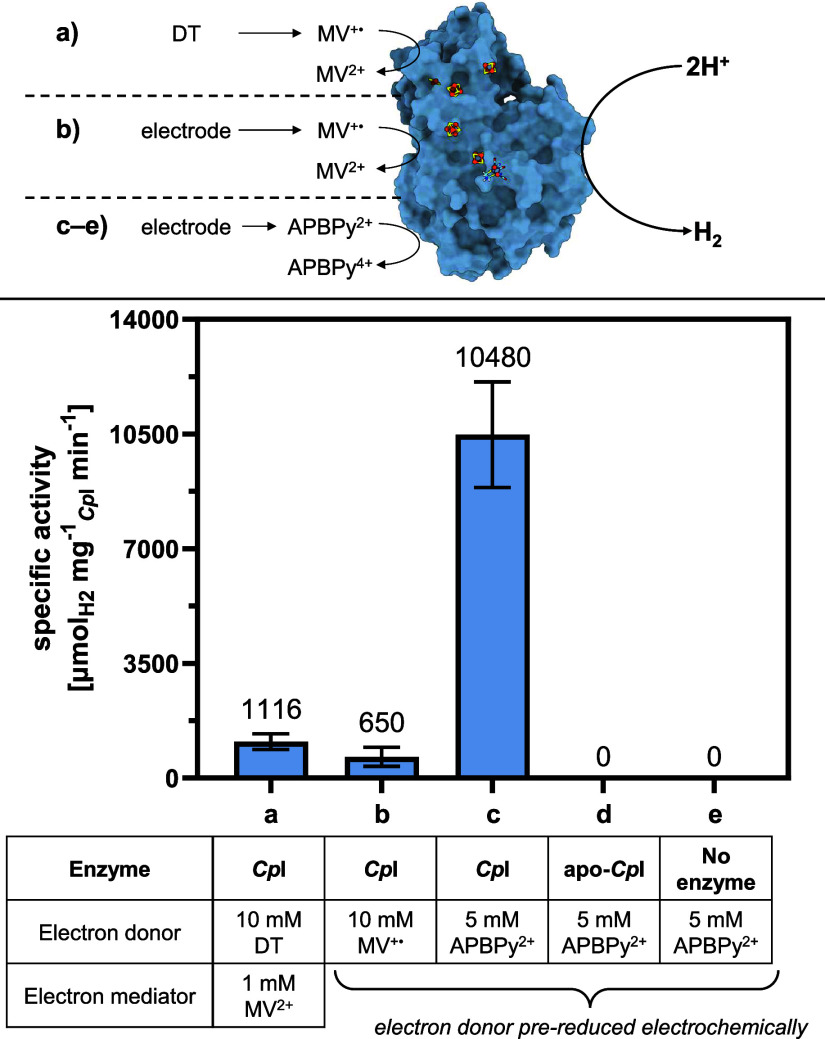
Top: electron transfer events for the different electron donor (and mediator) systems throughout this study. The [FeFe]-hydrogenase *Cp*I is shown, PDB 6N59. Bottom: specific activities of *Cp*I, apo-*Cp*I, and no enzyme with different electron donors/mediators at equimolar electron concentrations (10 ng/mL enzyme, 0.02 M TRIS pH 8, 10 mM electrons, 5 min, 37 °C).

The compatibility of [**APBPy**]^2+^ with different [FeFe]-hydrogenases was also evaluated to determine whether [**APBPy**]^2+^ could be employed more universally to increase the specific activities of other [FeFe]-hydrogenases under similar assay conditions. The hydrogenase from *Chlamydomonas reinhardtii* (*Cr*HydA1) structurally differs from *Cp*I in that it lacks all accessory FeS clusters and only contains the catalytically active H-cluster metallocofactor. In comparison to *Cp*I, *Cr*HydA1 has a maximum specific activity for H_2_ evolution of 935 μmol_H_2__ mg_*Cr*HydA1_^–1^ min^–1^ (748 μmol_H_2__ μmol_*Cp*I_^–1^ s^–1^, assuming a molecular mass of 48 kDa) using DT/MV as the electron donor system.^33^ *Cr*HydA1 heterologously produced in *E. coli* yielded a specific activity of 330 ± 37 μmol_H_2__ mg_*Cr*HydA1_^–1^ min^–1^ (400 ng mL^–1^ enzyme, 0.1 M DT, 0.01 M MV, 0.1 M MOPS, pH 7, 37 °C; Figure S16), which is comparable to reported values for the enzyme expressed in *E. coli*.^31,34^ The use of 0.02 M TRIS buffer at pH 8 lowered the activity with DT/MV by approximately 75% (85 ± 8 μmol_H_2__ mg_*Cr*HydA1_^–1^ min^–1^; Figure S16), consistent with the reported studies of the specific activity dependency on pH.^33^ Upon employing 5 mM [**APBPy**]^2+^ instead of DT/MV as the electron donor, an activity of 47 ± 14 μmol_H_2__ mg_*Cr*HydA1_^–1^ min^–1^ was observed (Figure S16), indicating that [**APBPy**]^2+^ is a relatively poorer electron donor to *Cr*HydA1 than it is to *Cp*I. It is possible that the high reducing power of [**APBPy**]^2+^ might irreversibly over-reduce the H-cluster and lower the specific activity of the enzyme, as has been observed by the group of Léger.^35^ This may be due to a “redox buffering” effect offered by the accessory FeS clusters, although further investigation is required to determine why this may not be the case for *Cp*I.

## Conclusions

We show that the previously reported viologen derivative [**APBPy**]^4+^, containing an additional phenylene bridge between the two pyridinium functionalities and *N*-alkylated propylammonium side chains, effectively functions as a low-potential electron donor to the [FeFe]-hydrogenase enzyme *Cp*I. The highly cationic nature of [**APBPy**]^4+^ with an overall charge of +4 maintains the solubility of the two-electron-reduced species in water, thus making a two-electron storage mediator accessible and effectively halving the concentration of the donor required in solution to achieve high enzymatic activity.

The decoupling of the pyridinium units results in a cathodic shift of *E* ^0^' in comparison to the first reduction potentials of parent MV^2+^, from −0.44 to −0.75 V *vs.* SHE, with both one-electron reductions taking place at almost the same *E* ^0^'. Voltammetric, coulometric, and UV/Vis spectroscopic data identified a singly reduced radical cation [**APBPy**]^3+•^ being formed en route to [**APBPy**]^2+^, thus confirming that two consecutive one-electron reductions occur. The stability of this highly reducing electron donor in biologically relevant buffers at different pHs was monitored by UV/Vis spectroscopy and showed high stability over all tested buffer systems and within the neutral pH range (7.5–8.2), thereby confirming the versatility of this mediator.

In the case of [FeFe]-hydrogenase *Cp*I, the activity assay conditions for the use of [**APBPy**]^2+^ were optimized, ultimately resulting in a significant increase in the specific activity of the enzyme for H_2_ production. An activity of 10,480 ± 1,607 μmol_H_2__ mg_*Cp*I_^–1^ min^–1^ (11,179 μmol_H_2__ μmol_*Cp*I_^–1^ s^–1^) was observed, which is a marked increase in the highest specific activity of *Cp*I (5,500 μmol_H_2__ mg_*Cp*I_^–1^ min^–1^) reported to date.^30^ This activity represents an almost 10-fold increase when compared to the DT/MV system and an approximately 20-fold increase against electrochemically prepared MV^+•^ under identical conditions for [**APBPy**]^2+^.

Finally, the specific activity of another [FeFe]-hydrogenase, *Cr*HydA1, was evaluated in addition to *Cp*I to determine whether there is a universal increase in activity across different types of [FeFe]-hydrogenases in combination with [**APBPy**]^2+^. Interestingly, the specific activity of *Cr*HydA1 when using [**APBPy**]^2+^ was found to be half that of DT/MV. In accordance with reports on a reductively inactivated H-cluster state,^35^ we postulate that this may be due to such an over-reduction, although further work is required to test this hypothesis. We intend to evaluate the activity of [**APBPy**]^2+^ with other [FeFe]-hydrogenases in addition to other metalloenzymes.

## Experimental Section

### Synthesis of 1,1′-bis­(3-(trimethylammonium)-propyl)-4,4′-(1,4-phenylene)-bipyridinium Tetrachloride ([**APBPy**]­Cl_4_)

[**APBPy**]­Cl_4_ was synthesized according to a modified literature procedure.^18^ A solution of 4,4′-(1,4-phenylene)­bipyridine (0.50 g, 2.15 mmol, 1 equiv) and (3-bromopropyl)­trimethylammonium bromide (3.37 g, 12.9 mmol, 6 equiv) in DMF (50 mL) was heated at 100 °C for 48 h under an Ar atmosphere. The resulting precipitate was filtered, washed with DMF (2 × 20 mL) and MeCN (2 × 20 mL), and dried under vacuum to obtain 1,1′-bis­(3-(trimethylammonium)-propyl)-4,4′-(1,4-phenylene)­bipyridinium tetrabromide [**APBPy**]­Br_4_ as a white solid.

#### Anion Exchange from Br^–^ to Cl^–^

[**APBPy**]­Br_4_ was dissolved in a small amount of water, and 10× excess of KPF_6_, dissolved in a small amount of water, was added. After addition, the immediate precipitation of 1,1′-bis­(3-(trimethylammonium)-propyl)-4,4′-(1,4-phenylene)­bipyridinium tetra­(hexafluorophosphate) [**APBPy**]­(PF_6_)_4_ as a white solid could be observed. The suspension was further stirred for 15 min at room temperature, and the precipitate was filtered, washed with water (5 × 5 mL), and dried overnight. [**APBPy**]­(PF_6_)_4_ was dissolved in a small amount of MeCN, and a 10× excess of tetrabutylammonium chloride, dissolved in a small amount of MeCN, was added. The suspension was further stirred for 10 min at room temperature, and the resulting off-white solid was filtered and washed with MeCN (5 × 5 mL). After recrystallization from H_2_O/DMF, the product (0.80 g, 1.38 mmol, 64%) was obtained as a crystalline white solid. The spectroscopic data are in agreement with reported values.^20^

^1^H NMR (400 MHz, DMSO-d_6_) δ [ppm] = 9.39 (d, *J* = 5.61 Hz, 4H), 8.78 (d, *J* = 5.61 Hz, 4H), 8.42 (s, 4H), 4.78 (t, *J* = 7.36 Hz, 4H), 3.52 (m, 4H), 3.13 (s, 18H), 2.54 (m, 4H).

^13^C­{^1^H}-NMR (100 MHz, DMSO-d_6_) δ [ppm] = 153.5, 145.4, 136.6, 129.4, 125.0, 61.8, 57.0, 52.5, 24.4.

### Electrosynthesis of [**APBPy**]^2+^

A 25 mM stock solution of [**APBPy**]­Cl_4_ was prepared in aqueous 0.5 M NaCl and added to an electrochemical cell. The cell was equipped with a stirring bar, a graphite rod as the working electrode (WE), a coiled Pt wire as the counter electrode (CE) in a separately fritted compartment containing the same electrolyte solution as the cell, and a Ag/AgCl (3 M) as the reference electrode (RE). CV was performed to ensure adequate conductivity and determine the exact reduction potential. Typically, a potential of approximately −0.85 V *vs*. SHE was applied under vigorous stirring of the solution. The color during prolonged chronoamperometry changed from colorless to yellow/brown to deep purple (Figure S5, bottom). Amperometry was stopped when the current plateaued to capacitive current (Figure S5, top).

### Enzyme Purification

The procedures for the expression and purification of the enzymes *Cp*I and *Cr*HydA1 were developed previously^31,36,37^ and can be found in detail in the Supporting Information.

### Enzyme Activity Measurements

Buffers were pH-adjusted using HCl/NaOH and deoxygenated by applying three cycles of vacuum and N_2_, before being brought into an Ar glovebox and stirred overnight. Activity assays for proton reduction were performed in 13 mL Wheaton vials. Buffer and enzyme were mixed in a glovebox on ice. The respective electron donor was diluted to a total volume of 1 mL. The samples were sealed under Ar and brought out of the glovebox for incubation at a specified temperature and time (see the respective experiments for the specifications). After incubation, the samples were brought back to 0 °C, and the headspace was sampled with a syringe (VICI, Pressure-Lok). The syringe was vented to atmospheric pressure and injected into a gas chromatograph (SRI 8610C Gas Chromatograph, SRI Instruments, California) equipped with a thermal conductivity detector for quantification. Samples were quantified by comparison to a previously recorded calibration curve (Figure S15), where known amounts of H_2_ were added to sample vials under the same experimental conditions.

## Supplementary Material



## Data Availability

The data underlying this study are available in the published article, in its Supporting Information, and openly available in Zenodo at DOI: 10.5281/zenodo.15688676.

## Footnotes

1 Only the first reduction events of MV^2+^ and TQ^2+^ are considered since the two-electron reduced forms are inaccessible in aqueous media due to the insolubility of neutral MV^0^ and TQ^0^, as can be seen by the respective CVs in water (Figure S7).

2 MOPS: 3-(morpholin-4-yl)­propane-1-sulfonic acid; HEPES: 2-[4-(2-hydroxyethyl)­piperazin-1-yl]­ethane-1-sulfonic acid; TRIS: 2-amino-2-(hydroxymethyl)­propan-1,3-diol; TAPS: *N*-tris­(hydroxymethyl)­methyl-3-aminopropanesulfonic acid.

3 A TOF of 21,000 ± 12,000 s^–1^ has been proposed for H_2_ evolution by [FeFe]-hydrogenase from *Clostridium acetobutylicum* (*Ca*HydA) although this value is obtained by extrapolating out an expected exponential increase in *k*_ET_ with decreasing distance between the enzyme and an electrode suface to a theoretical minimum distance.^28^

## Notes

Experimental details about the synthesis of [**APBPy**]^4+^ and the expression of *Cp*I and *Cr*HydA1; electrochemical and spectroscopic measurements; activity assays using electrochemically reduced [**APBPy**]^2+^ as well as the real-time mass-spectrometric measurements; additional references^38^ (PDF)
