# Supplementary material for: A Low-Potential π‑Extended Viologen Electron Donor Results in Increased H2 Production by CpI [FeFe]-Hydrogenase
Source: ACS Org Inorg Au. 2026 Jan 20;6(4):398–405. doi: 10.1021/acsorginorgau.5c00106 (PMC13449430; doi:10.1021/acsorginorgau.5c00106)
Supplement: Supplementary file 1 [file gg5c00106_si_001.pdf]

Supporting Information: A Low-potential π-extended Viologen Electron Donor Results in Increased H_2 Production by CpI [FeFe]-hydrogenase

Nils Ostermann,[*,†,‡] Sophie Webb,[†,‡] Andrea Do Nascimento Henriques,[†,‡] and Ross D. Milton[*,†,‡]

†*University of Geneva, Department of Inorganic and Analytical Chemistry, Sciences II, Quai Ernest-Ansermet 30, 1211 Geneva 4, Switzerland*

‡*National Centre of Competence in Research (NCCR) Catalysis, University of Geneva, 1211 Geneva 4, Switzerland*

E-mail: nils.ostermann@unige.ch; ross.milton@unige.ch

Table of Contents

General Considerations 4

Electrochemical and Spectroscopic Measurements 5

Enzyme Purification 5

Real-Time Mass Spectrometry 6

Additional NMR Spectra 7

Additional Electrochemical Data 9

Additional UV/vis Spectra 12

Additional [FeFe]-hydrogenase Activity Data 14

List of Figures

S1 SDS-PAGE (10% acrylamide, Bis-Tris) of *Cp*I, apo-*Cp*I and *Cr*HydA1 (4 μg enzyme/well) used in this study (Thermoscientific PageRuler Prestained Protien Ladder), 120 V. 6

S2 ^{1}H-NMR spectrum of [**APBPy**]Cl_4 in DMSO-d_6 (note that the peak at 2.54 ppm is only half integrated, due to the overlap with the solvent peak), 400 MHz. 7

S3 ^{13}C{^{1}H}-NMR spectrum of [**APBPy**]Cl_4 in DMSO-d_6, 100 MHz. 8

S4 DOSY-NMR spectrum of [**APBPy**]Cl_4 in D_2O, 400 MHz. 8

S5 Top: representative i/t (black) and Q/t curves (blue) upon bulk chronoamperometry of [**APBPy**]Cl_4 in aqueous NaCl (0.5 M), c = 25 mM, $E_{\mathrm{applied}} = -0.85$ V *vs.* SHE; bottom: photographs of the color change during prolonged amperometry. 9

S6 Left: scanrate dependent CVs of 0.5 mM [**APBPy**]$^{4+}$ in aqueous NaCl (0.1 M), GC working electrode, Pt counter electrode, SCE reference electrode (converted to SHE by applying the conversion factor of +0.241 V), plotted in IUPAC convention. Right: current density *vs.* square root of the scanrate for $E_{\mathrm{p,c}} = -0.77$ V (black) and $E_{\mathrm{p,a}} = -0.72$ V (red). 9

S7 CVs of MV^{2+} (left) and TQ^{2+} (right) in aqueous NaCl (0.1 M), c = 5 mM, $v = 2.5$ mVs^{-1}, GC working electrode, Pt counter electrode, SCE reference electrode (converted to SHE by applying the conversion factor of +0.241 V), plotted in IUPAC convention. 10

S8 Concentration dependent CVs (left, v = 50 mV/s) and DPVs (right, v = 5 mV/s, modulation amplitude = 25 mV, modulation time = 10 ms, interval time = 0.1 s) of [**APBPy**]$^{4+}$ in aqueous NaCl (0.1 M), GC working electrode, Pt counter electrode, SCE reference electrode (converted to SHE by applying the conversion factor of +0.241 V), plotted in IUPAC convention. 10

S9 Concentration dependent CVs (left, v = 2.5 mV/s) and DPVs (right, v = 5 mV/s, modulation amplitude = 25 mV, modulation time = 10 ms, interval time = 0.1 s) of [**APBPy**]$^{4+}$ in aqueous TRIS (0.02 M, pH 8), GC working electrode, Pt counter electrode, SCE reference electrode (converted to SHE by applying the conversion factor of +0.241 V), plotted in IUPAC convention. 11

S10 CVs of [**APBPy**]$^{4+}$ (0.1 mM, black) and after addition of *Cp*I (0.4 mg/mL, blue) in TRIS (0.02 M, pH 8) at s scanrate of 2.5 mVs^{-1}, GC working electrode, Pt counter electrode, SCE reference electrode (converted to SHE by applying the conversion factor of +0.241 V), plotted in IUPAC convention. 11

S11 Left: UV/vis spectra of [**APBPy**]$^{2+}$ in H_2O, c from 0.004 mM (black) to 0.026 mM (light blue). Right: Absorbance at 544 nm *vs.* the concentration of [**APBPy**]$^{2+}$. 12

S12 Top: UV/vis spectra in H_2O upon addition of equimolar [**APBPy**]$^{2+}$ to [**APBPy**]$^{4+}$ (left) and addition of equimolar [**APBPy**]$^{4+}$ to [**APBPy**]$^{2+}$ (right). c_{total} ≈0.02 mM (red), 0.03 mM (green), 0.04 mM (blue). Initial spectra, before mixing, in the respective faint color. 12

S13 Stability of electrochemically prepared [**APBPy**]$^{2+}$ ($c \approx$ 0.01 mM) in 0.01 M TRIS buffer at different pH (blue: 8.12; orange: 8.00; pink: 7.55) and in unbuffered MilliQ water as comparison (black). Visible absorbance at 510 nm (left) and 544 nm (right) monitored over time. 13

S14 Stability of electrochemically prepared [**APBPy**]$^{2+}$ ($c \approx 0.01$ mM) in different buffers, 0.01 M, pH 8 (red: HEPES; orange: phosphate; blue: TRIS; green: MOPS; purple: TAPS) and in unbuffered MilliQ water as comparison (black). Visible absorbance at 510 nm (left) and 544 nm (right) monitored over time. 13
S15 Representative GC calibration curve for quantitative H_2 detection. 14
S16 Left: standard specific activities of *Cp*I and *Cr*HydA1 in MOPS (0.1 M, pH 7) with DT (0.1 M) and MV (0.01 M) as electron donor/mediator (400 ng/mL enzyme, 20 min, 37 °C); right: specific activities of *Cr*HydA1 in TRIS (0.02 M, pH 8) with DT (0.1 M)/MV (0.01 M) and [**APBPy**]$^{2+}$ (5 mM), respectively (400 ng/mL enzyme, 5 min, 37 °C). 14
S17 Partial pressure of H_2 *vs.* time from real-time MS measurements of *Cp*I activity assays. Blue trace: 400 ng/mL enzyme in TRIS (0.02 M, pH 8) with DT (0.1 M) and MV (0.01 M) as electron donor/mediator (400 ng/mL enzyme, 37 °C). Black trace: [**APBPy**]$^{2+}$ (5 mM) as electron donor in TRIS (0.02 M, pH 8, 40 ng/mL enzyme, room temperature). Inlet: Zoom into the first 12 min of both experiments, to see initial rates. 15
S18 Partial pressure of H_2 *vs.* time from a real-time MS measurement of *Cp*I with [**APBPy**]$^{2+}$ (5 mM) as electron donor in TRIS (0.02 M, pH 8, 10 ng/mL enzyme, room temperature). 15

General Considerations

Commercially avaialble 4,4'-(1,4-phenylene)bispyridine (ABCR, Fluorochem), (3-bromopropyl)-trimethylammonium bromide (Fluorochem), KPF_6 (Sigma-Aldrich), $^{n}Bu_4NCl$ (Sigma-Aldrich) and Methylviologen (Sigma-Aldrich) were used as purchased. Triquat was synthesized as reported.[1]

Electrochemical and Spectroscopic Measurements

All electrochemical experiments were performed in an Ar-filled glovebox (Jacomex, France) in an anoxic environment (O_2 concentration < 1 ppm) at room temperature. Electrochemical data were recorded using a Metrohm-Autolab potentiostat (PGSTAT302N) in a three-electrode setup with a 3 mm diameter glassy carbon (GC) disk (CH Instruments, CHI104) for voltammetry and a graphite rod (3 mm diameter) for chronoamperometry as the working electrode (WE). GC electrodes were polished on a polishing pad consisting of a α-Al_2O_3 (CH Instruments) slurry. A coiled Pt-wire in a separately fritted compartment containing the same buffer as the cell was used as the counter electrode (CE) and a saturated calomel electrode (SCE) (CH Instruments, CHI150) or Ag/AgCl (3 M) electrode was used as the reference electrode (RE). Potentials were converted *vs.* the standard hydrogen electrode (SHE) by applying a conversion factor (+0.241 V for SCE and +0.196 V for Ag/AgCl (3 M)).

NMR spectra were recorded with a Bruker Avance 400 MHz spectrometer at 298 K. Chemical shifts (in ppm) are reported with reference to the solvent signal (DMSO: $\delta_{1H} = 2.50$ ppm and $\delta_{13C} = 39.52$ ppm) and multiplicities denoted as s = singlet, d = dublet, t = triplet and m = multiplet.

UV/vis spectra were recorded with a Perkin Elmer Lambda 365 spectrophotometer in a quartz cuvette with a path length of 1 cm at 298 K.

Enzyme Purification

[FeFe]-hydrogenases from *Clostridium pasteurianum* (*Cp*I) and *Chlamydomonas reinhardtii* (*Cr*HydA1), both with C-terminal Strep-tags, were recombinantly expressed in *Escherichia coli* (*E. coli*) BL21(DE3)Δ*iscR*.[2] *Cp*I was expressed alongside the HydEFGX maturases from *Shewanella oneidensis*, as reported previously.[3,4] To purify the proteins, cells were resuspended in buffer (50 mM Tris:HCl, 150 mM NaCl, 4 mM sodium dithionite, pH 8), in 1.5% of the inital culture volume. The cells were subsequently lysed *via* sonication under

anoxic conditions (Ar atmosphere) and centrifuged (30'000 x g, 1 h) to pellet the insoluble components and cell debris. The supernatant was loaded on a StrepTactin-XT column (Cytiva, 5 mL, flow rate 5-10 mL min^{-1}) and the enzymes were eluted with 50 mM biotin (in 50 mM Tris:HCl and 150 mM NaCl, pH 8). The purified protein fraction was concentrated with a stirred concentration cell (Millipore, 30 kDa cut-off membrane for *Cp*I, 10 kDa cut-off membrane for *Cr*HydA1), flash frozen and stored in liquid nitrogen. The concentration and purity of the enzymes were determined *via* the Biuret method (using bovine serum albumin as a standard) and SDS-PAGE (precast mPAGE 10% Bis-Tris gels from Merck Millipore), respectively (Fig. S1). As given in Fig. S16, the specific activities of these enzymes under *standard conditions* for H_2 production were determined to be 1'968 ± 308 μmol_{H_2} ${mg_{CpI}}^{-1}$ min^{-1} for *Cr*HydA1 and 330 ± 37 μmol_{H_2} ${mg_{CpI}}^{-1}$ min^{-1} for *Cr*HydA1.

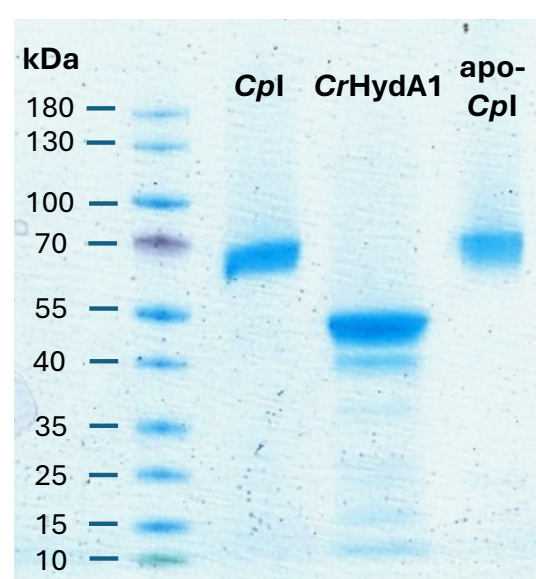

Figure S1: SDS-PAGE (10% acrylamide, Bis-Tris) of *Cp*I, apo-*Cp*I and *Cr*HydA1 (4 μg enzyme/well) used in this study (Thermoscientific PageRuler Prestained Protien Ladder), 120 V.

Real-Time Mass Spectrometry

Real-time mass spectrometric (RT-MS) measurements were performed in an Ar-filled Glovebox (Jacomex, France). Samples were prepared in 13 mL Wheaton vials and sealed air tight with butyl stoppers. The production of H_2 was monitored by a residual gas analyzer (HPR-20 R&D, HIDEN Analytical), equipped with an unheated capillary inlet. The inlet was connected air-tightly to the glovebox through a feedthrough and fed through the butyl stopper of the reaction vessel with a PEEK tube (0.13 mm diameter). An additional PEEK

tube was attached to the vial to relieve the cell from the vacuum generated by the RT-MS over time (open to the Ar glovebox environment).

Additional NMR Spectra

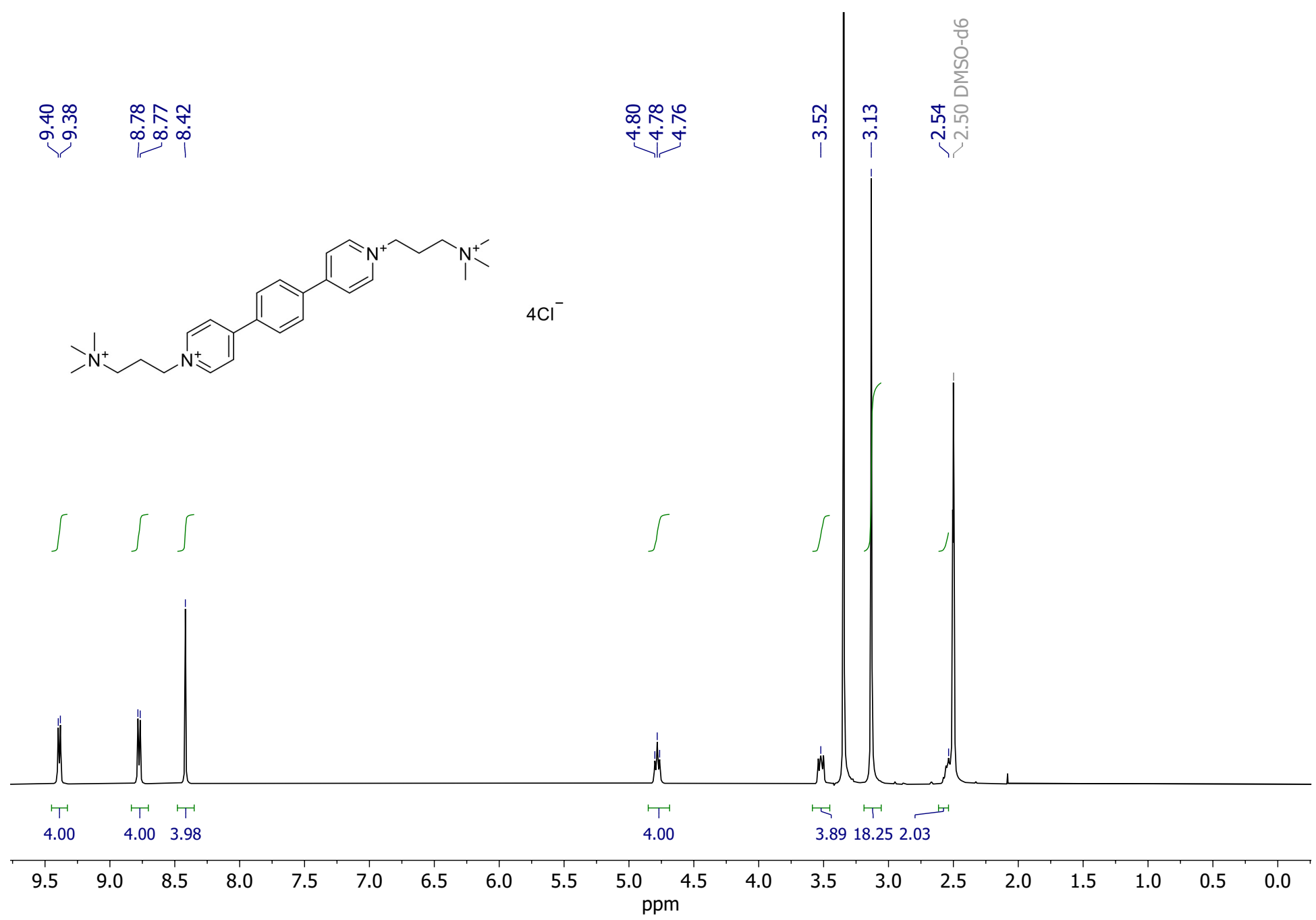

Figure S2: ^{1}H-NMR spectrum of [**APBPy**]Cl_4 in DMSO-d_6 (note that the peak at 2.54 ppm is only half integrated, due to the overlap with the solvent peak), 400 MHz.

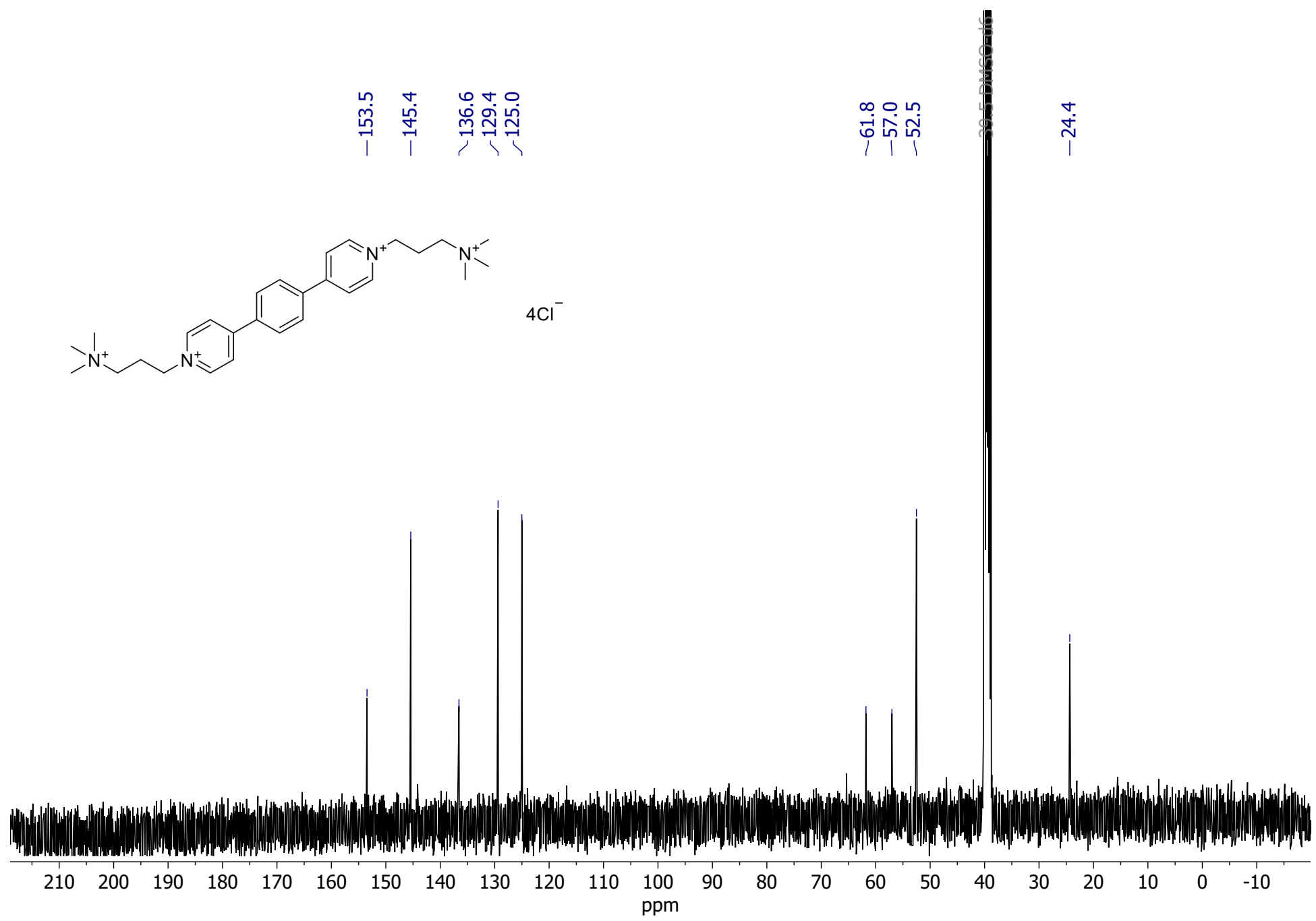

Figure S3: $^{13}C\{^{1}H\}$-NMR spectrum of [**APBPy**]Cl_4 in DMSO-d_6, 100 MHz.

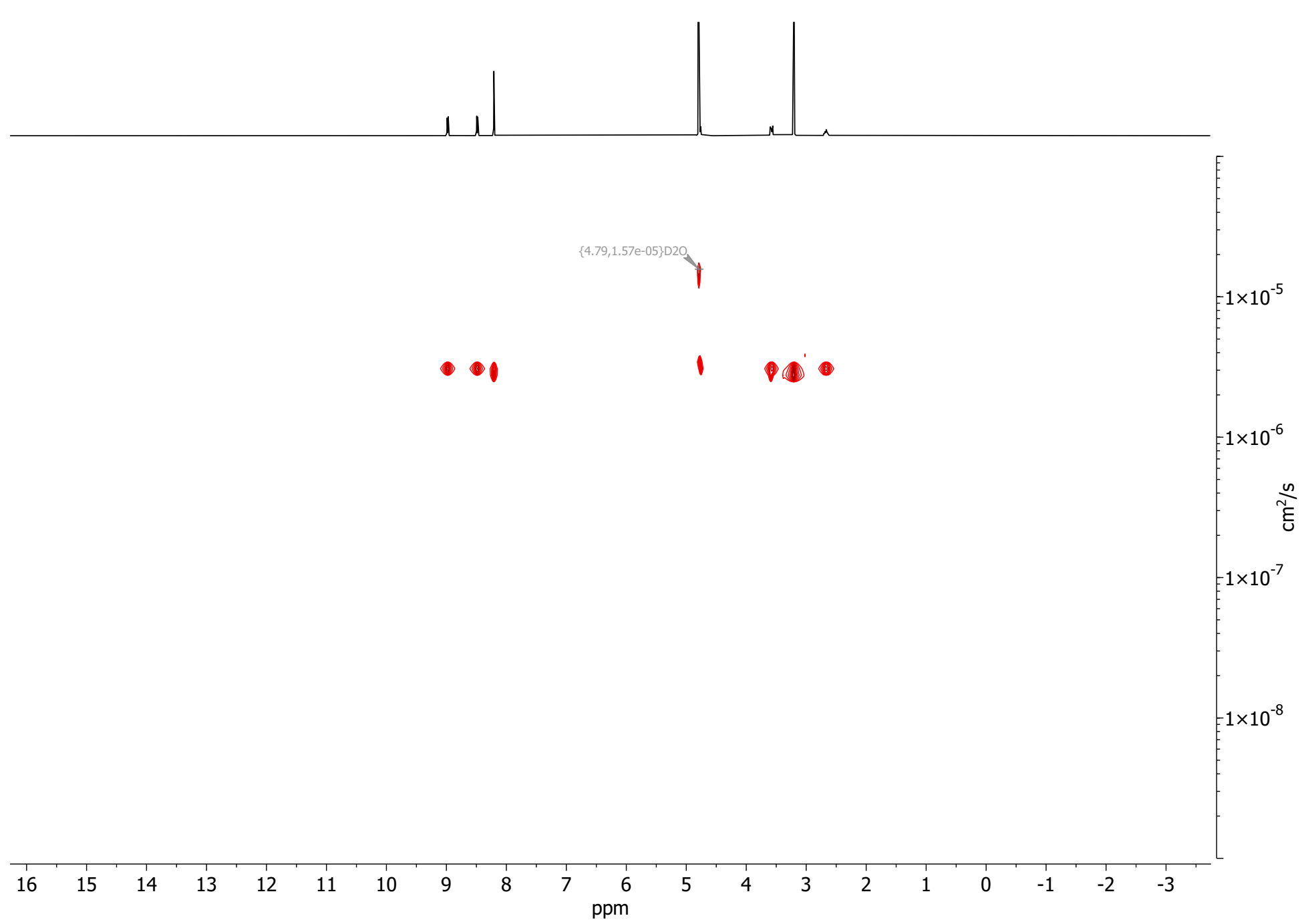

Figure S4: DOSY-NMR spectrum of [**APBPy**]Cl_4 in D_2O, 400 MHz.

Additional Electrochemical Data

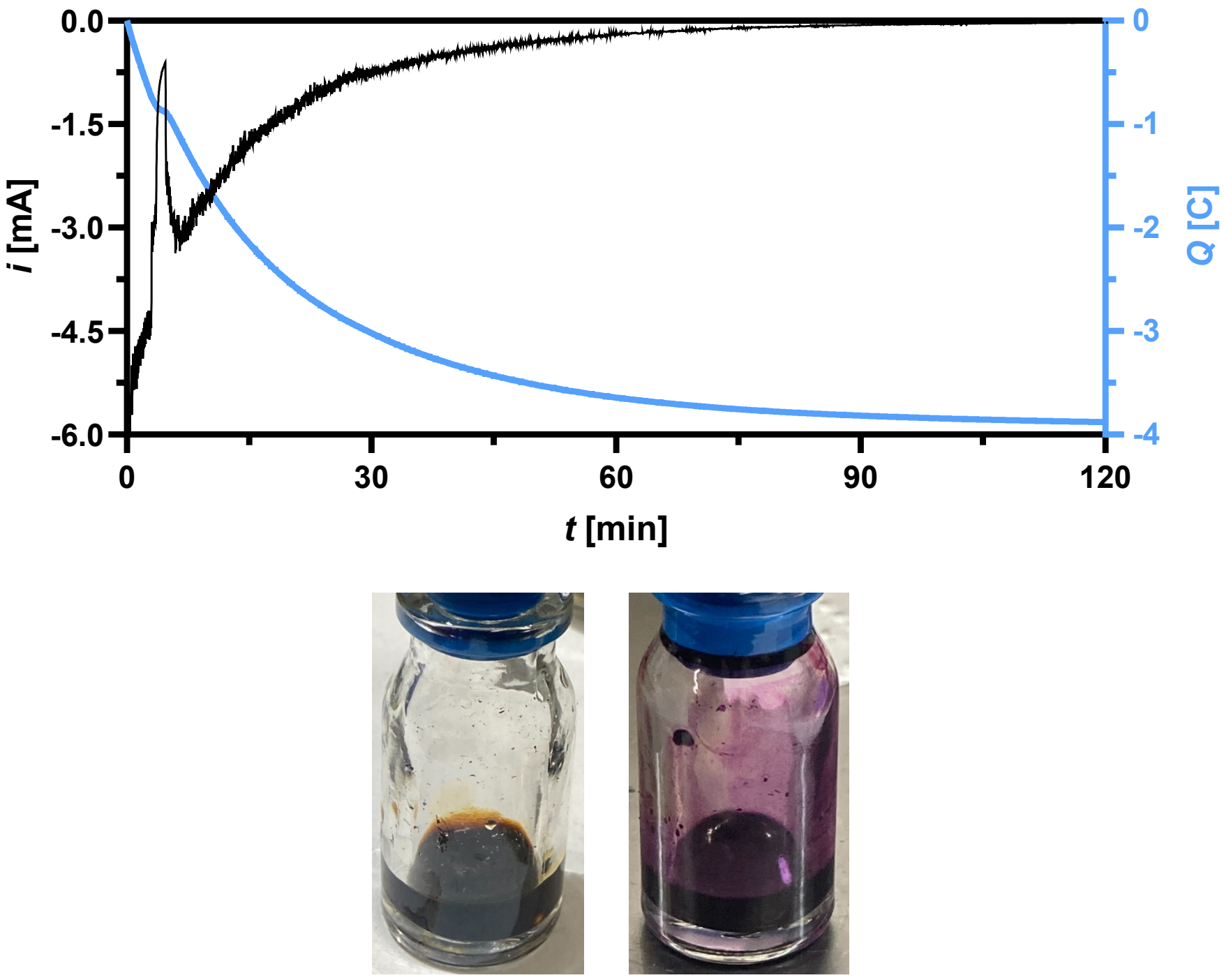

Figure S5: Top: representative i/t (black) and Q/t curves (blue) upon bulk chronoamperometry of [**APBPy**]Cl_4 in aqueous NaCl (0.5 M), $c = 25$ mM, $E_{\text{applied}} = -0.85$ V *vs.* SHE; bottom: photographs of the color change during prolonged amperometry.

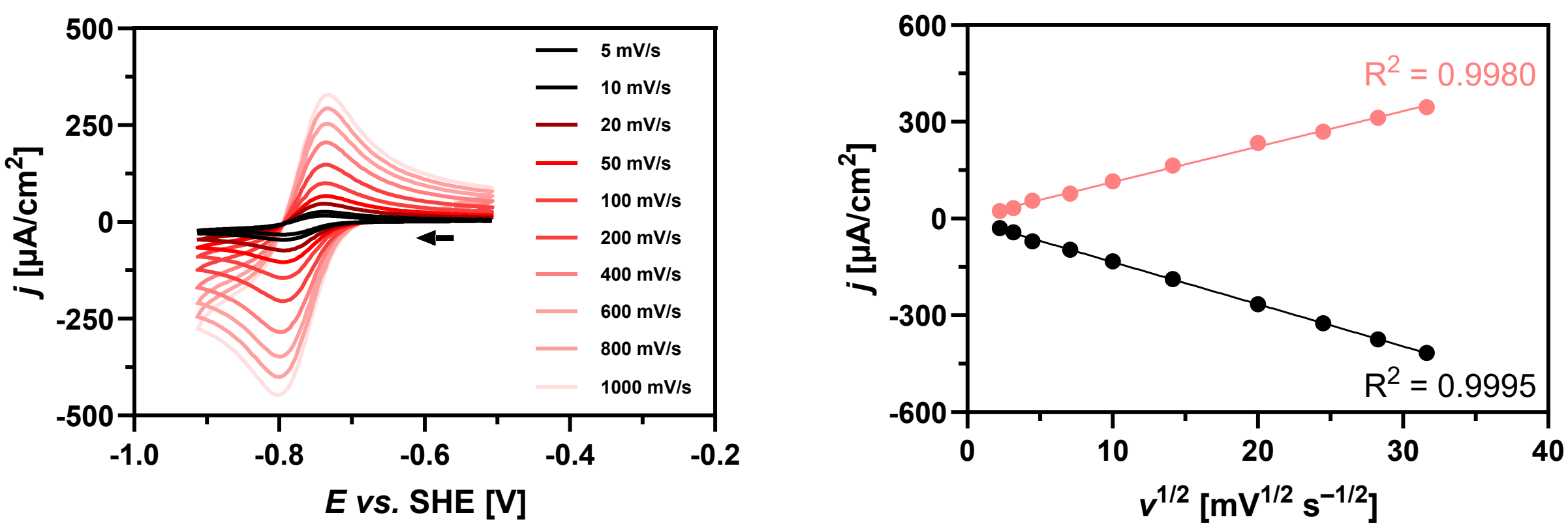

Figure S6: Left: scanrate dependent CVs of 0.5 mM [**APBPy**]$^{4+}$ in aqueous NaCl (0.1 M), GC working electrode, Pt counter electrode, SCE reference electrode (converted to SHE by applying the conversion factor of +0.241 V), plotted in IUPAC convention. Right: current density *vs.* square root of the scanrate for $E_{\text{p,c}} = -0.77$ V (black) and $E_{\text{p,a}} = -0.72$ V (red).

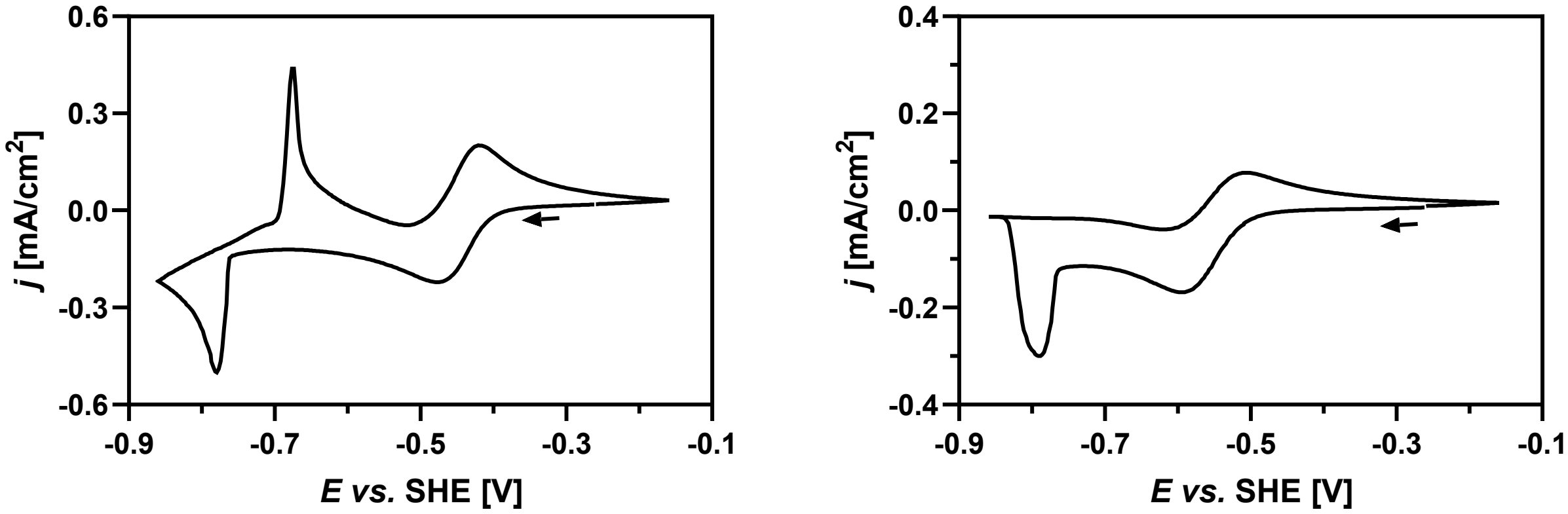

Figure S7: CVs of MV^{2+} (left) and TQ^{2+} (right) in aqueous NaCl (0.1 M), c = 5 mM, v = 2.5 mVs^{-1}, GC working electrode, Pt counter electrode, SCE reference electrode (converted to SHE by applying the conversion factor of +0.241 V), plotted in IUPAC convention.

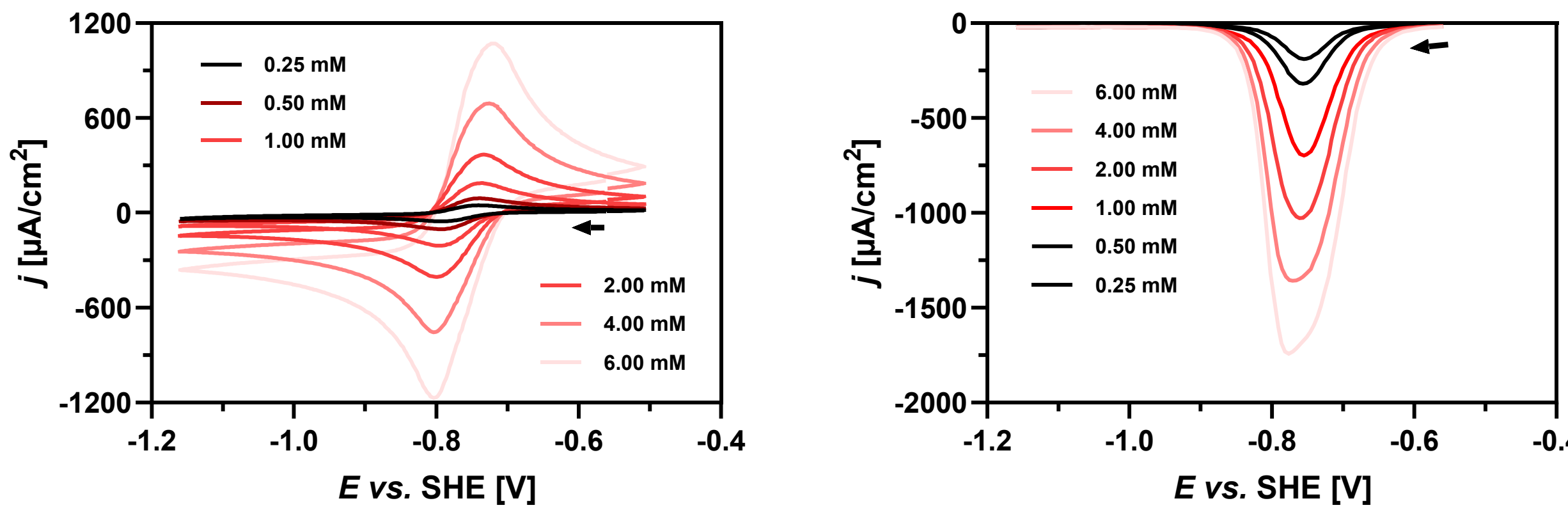

Figure S8: Concentration dependent CVs (left, v = 50 mV/s) and DPVs (right, v = 5 mV/s, modulation amplitude = 25 mV, modulation time = 10 ms, interval time = 0.1 s) of [**APBPy**]$^{4+}$ in aqueous NaCl (0.1 M), GC working electrode, Pt counter electrode, SCE reference electrode (converted to SHE by applying the conversion factor of +0.241 V), plotted in IUPAC convention.

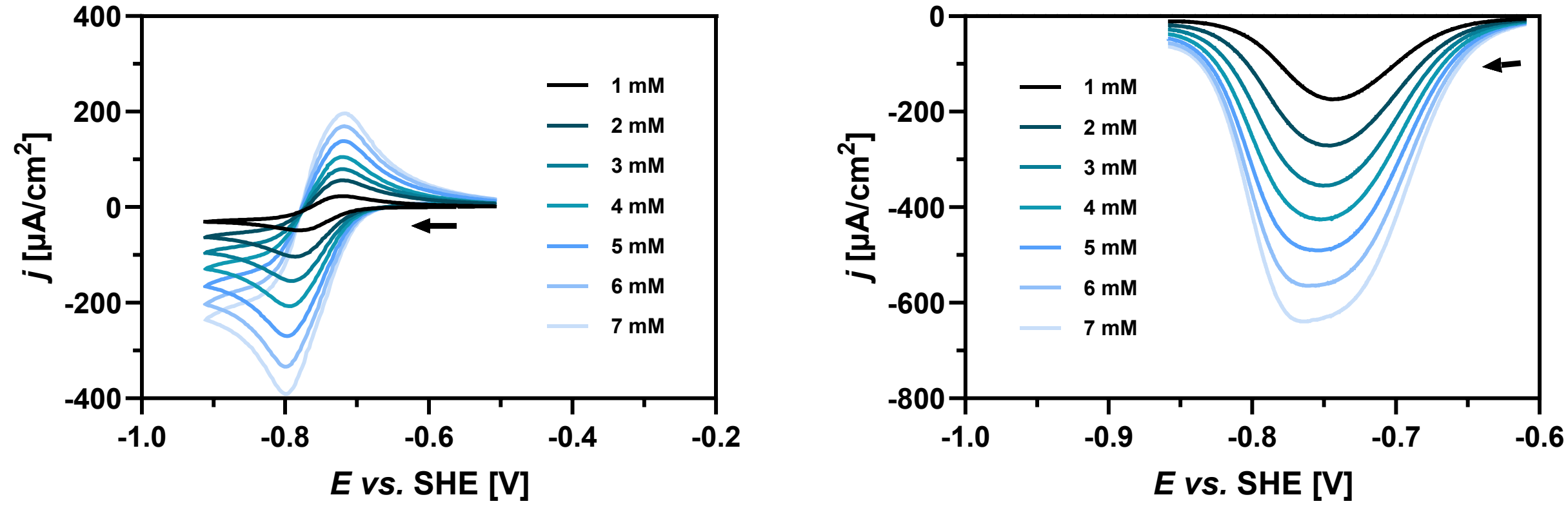

Figure S9: Concentration dependent CVs (left, v = 2.5 mV/s) and DPVs (right, v = 5 mV/s, modulation amplitude = 25 mV, modulation time = 10 ms, interval time = 0.1 s) of [**APBPy**]$^{4+}$ in aqueous TRIS (0.02 M, pH 8), GC working electrode, Pt counter electrode, SCE reference electrode (converted to SHE by applying the conversion factor of +0.241 V), plotted in IUPAC convention.

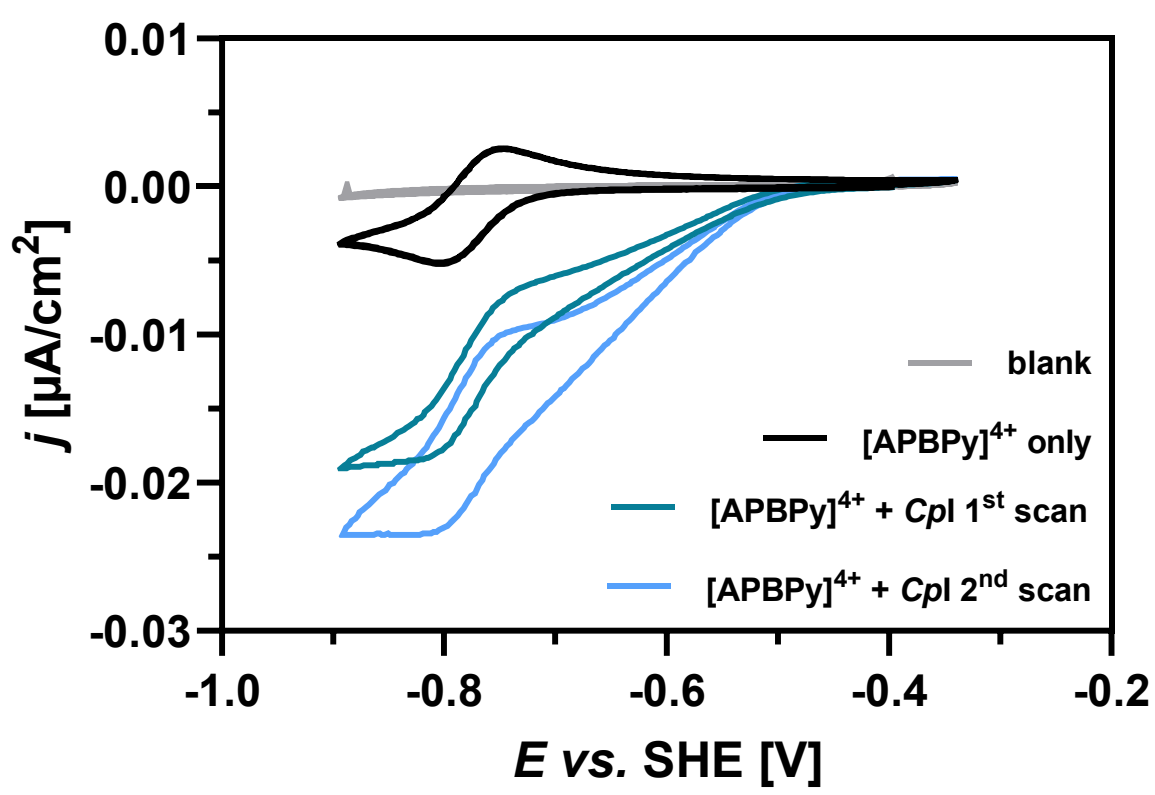

Figure S10: CVs of [**APBPy**]$^{4+}$ (0.1 mM, black) and after addition of *Cp*I (0.4 mg/mL, blue) in TRIS (0.02 M, pH 8) at s scanrate of 2.5 mVs^{-1}, GC working electrode, Pt counter electrode, SCE reference electrode (converted to SHE by applying the conversion factor of +0.241 V), plotted in IUPAC convention.

Additional UV/vis Spectra

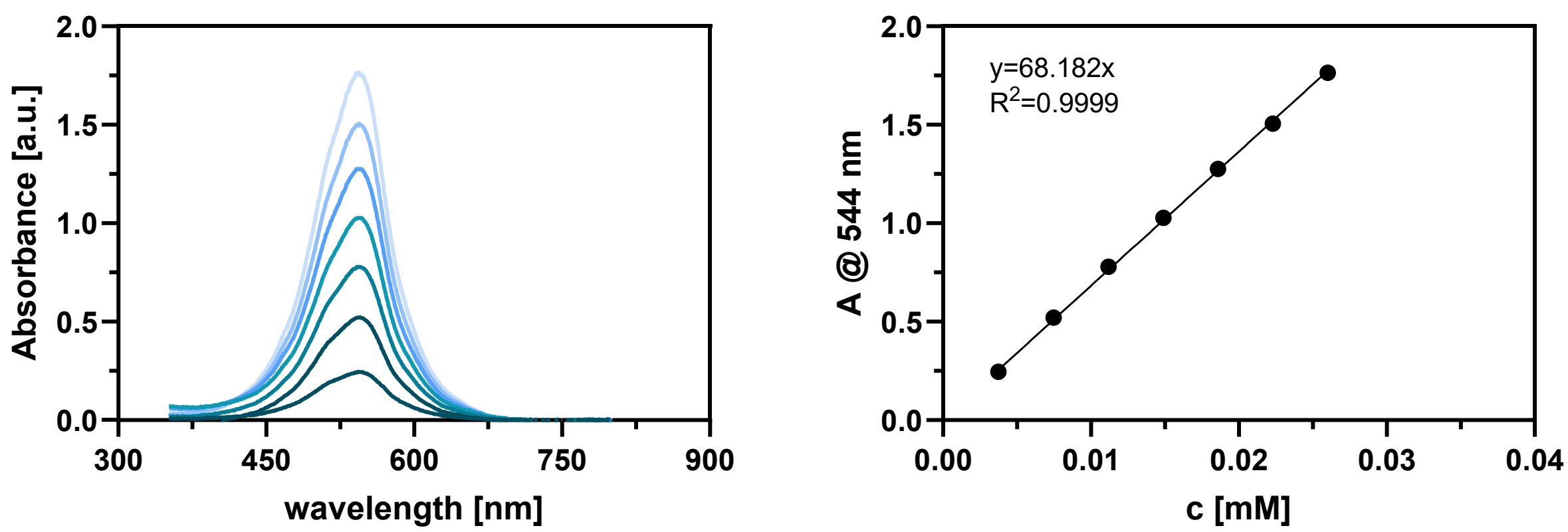

Figure S11: Left: UV/vis spectra of [**APBPy**]$^{2+}$ in H_2O, c from 0.004 mM (black) to 0.026 mM (light blue). Right: Absorbance at 544 nm *vs.* the concentration of [**APBPy**]$^{2+}$.

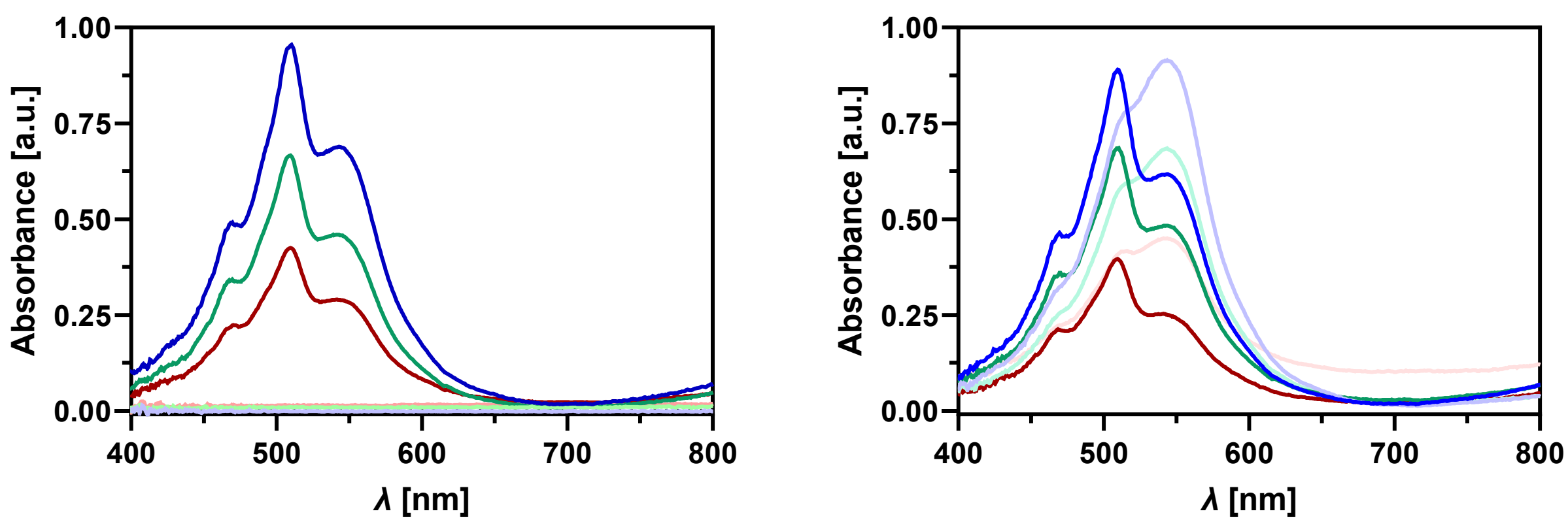

Figure S12: Top: UV/vis spectra in H_2O upon addition of equimolar [**APBPy**]$^{2+}$ to [**APBPy**]$^{4+}$ (left) and addition of equimolar [**APBPy**]$^{4+}$ to [**APBPy**]$^{2+}$ (right). c_{total} ≈0.02 mM (red), 0.03 mM (green), 0.04 mM (blue). Initial spectra, before mixing, in the respective faint color.

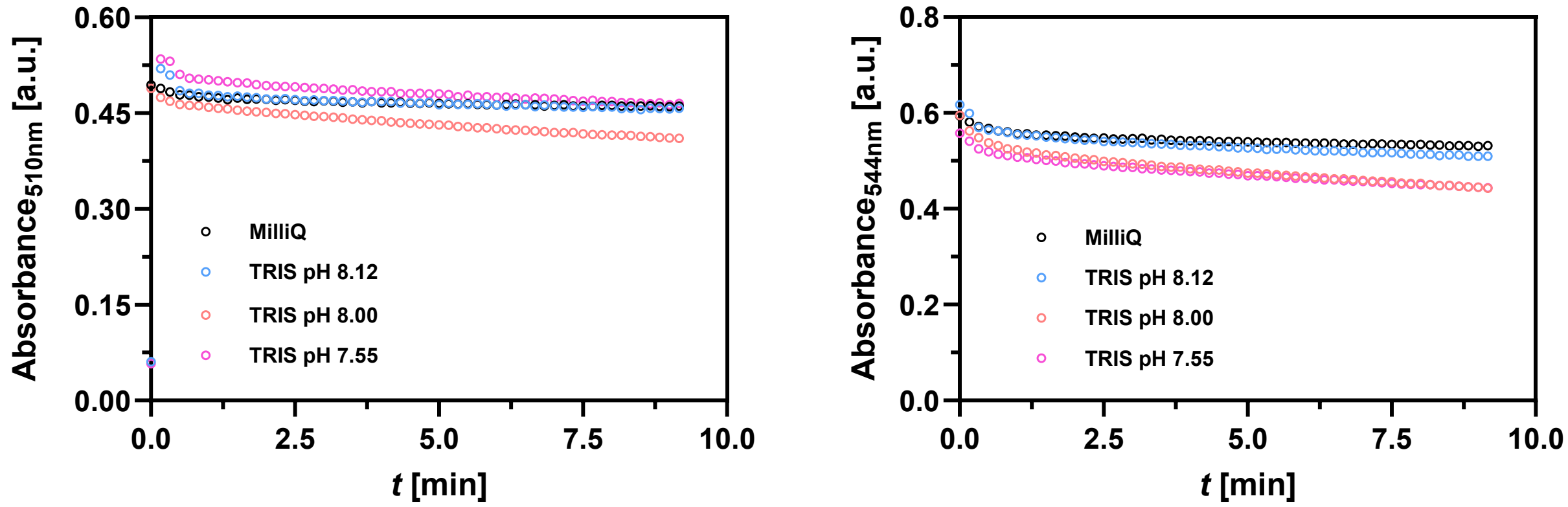

Figure S13: Stability of electrochemically prepared [**APBPy**]$^{2+}$ ($c \approx$ 0.01 mM) in 0.01 M TRIS buffer at different pH (blue: 8.12; orange: 8.00; pink: 7.55) and in unbuffered MilliQ water as comparison (black). Visible absorbance at 510 nm (left) and 544 nm (right) monitored over time.

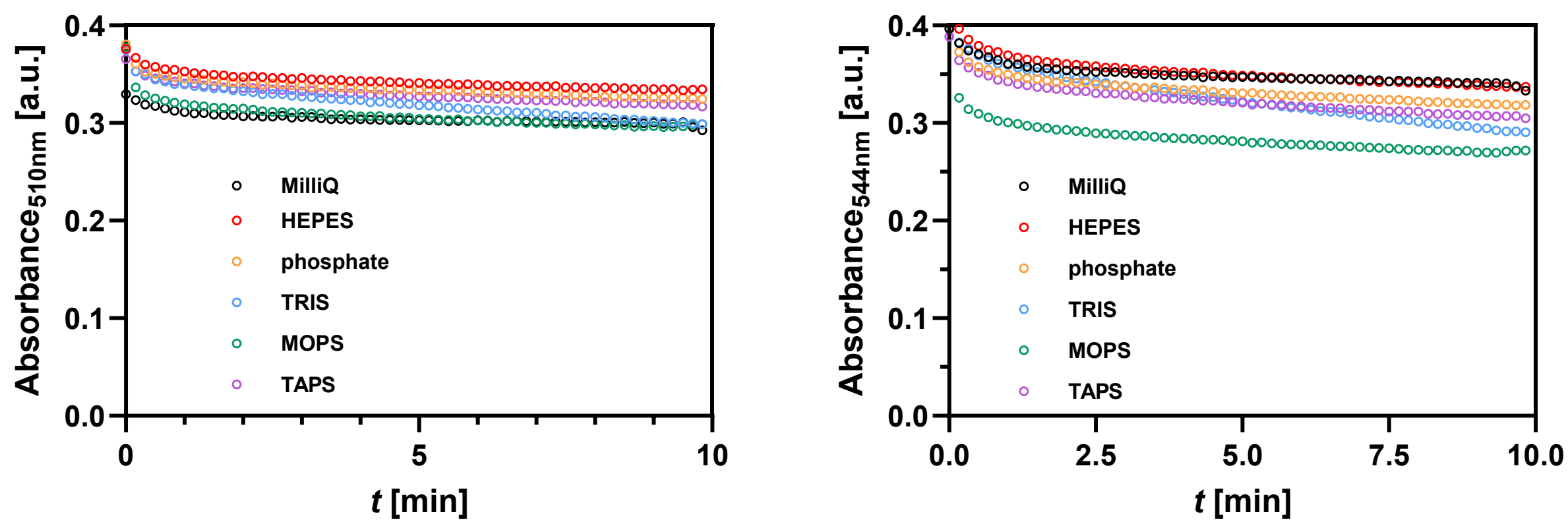

Figure S14: Stability of electrochemically prepared [**APBPy**]$^{2+}$ ($c \approx$ 0.01 mM) in different buffers, 0.01 M, pH 8 (red: HEPES; orange: phosphate; blue: TRIS; green: MOPS; purple: TAPS) and in unbuffered MilliQ water as comparison (black). Visible absorbance at 510 nm (left) and 544 nm (right) monitored over time.

Additional [FeFe]-hydrogenase Acitivty Data

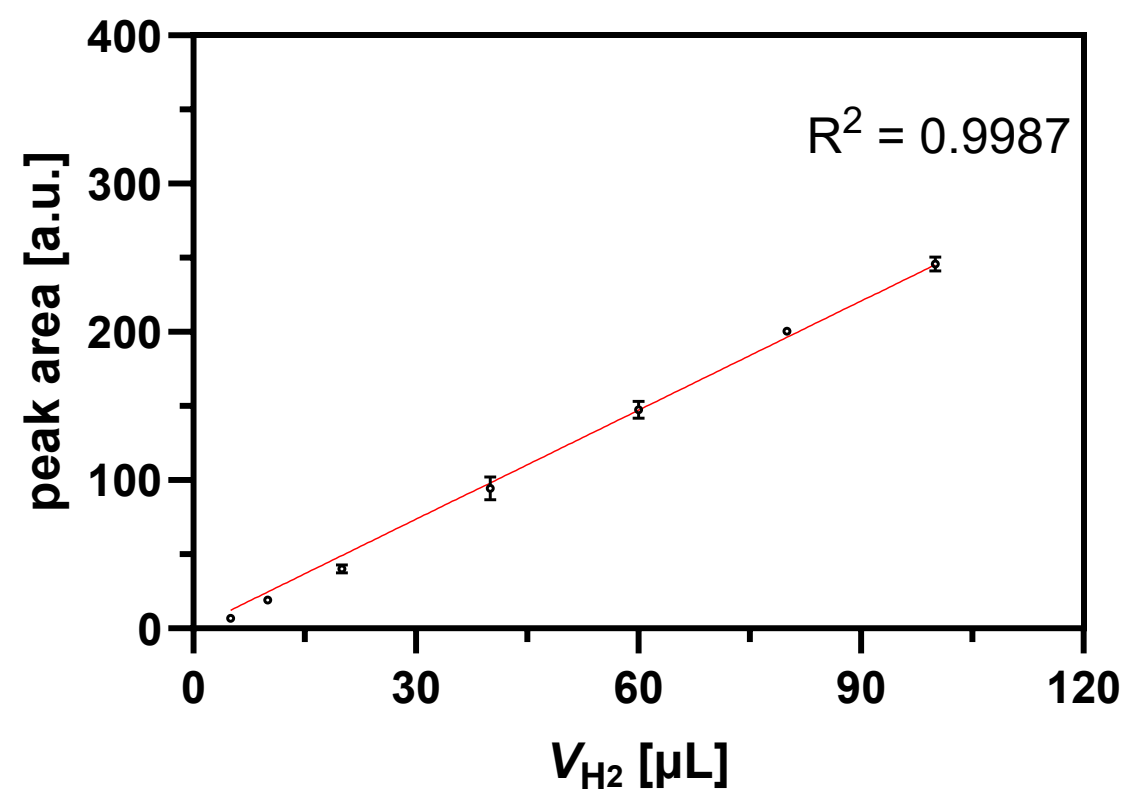

Figure S15: Representative GC calibration curve for quantitative H_2 detection.

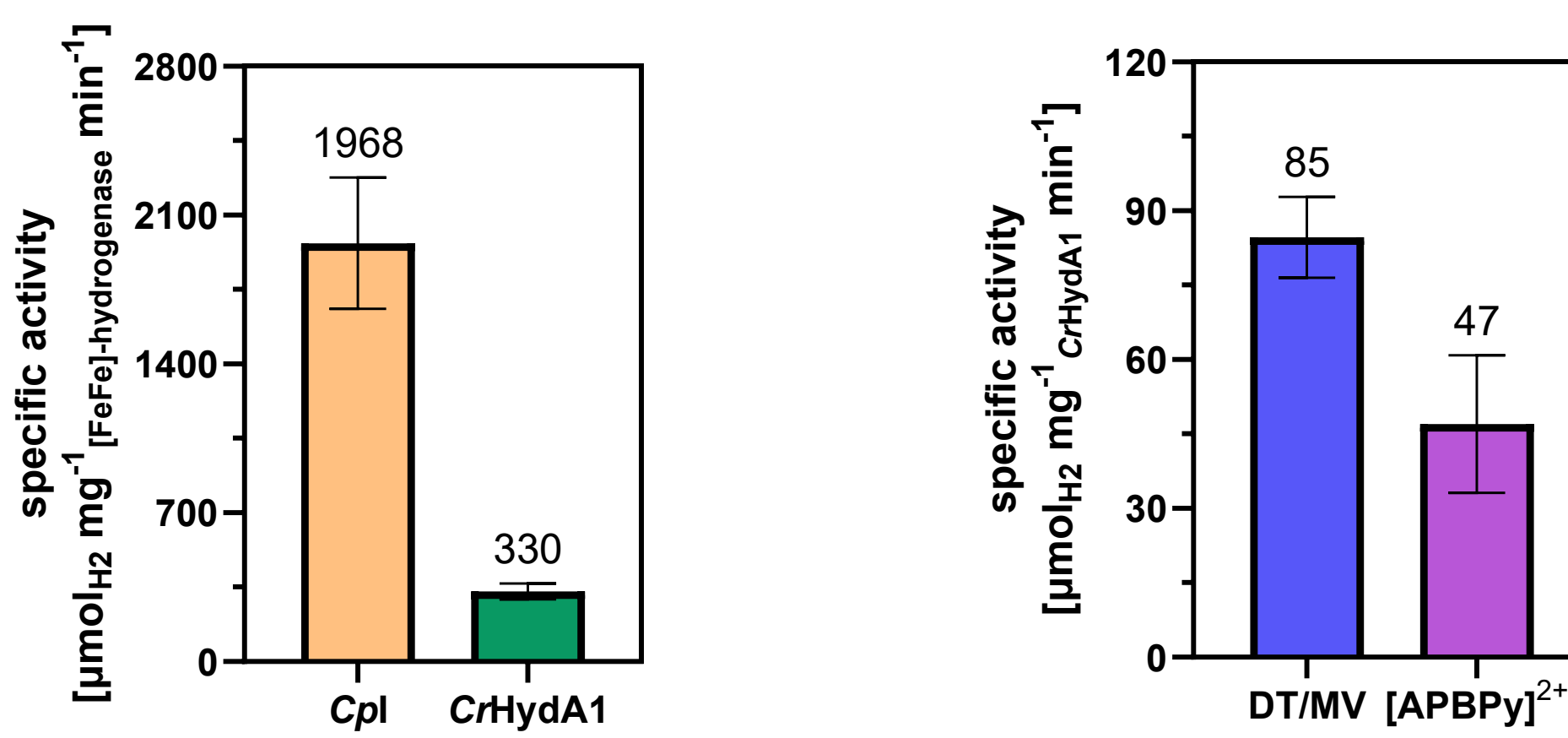

Figure S16: Left: standard specific activities of *Cp*I and *Cr*HydA1 in MOPS (0.1 M, pH 7) with DT (0.1 M) and MV (0.01 M) as electron donor/mediator (400 ng/mL enzyme, 20 min, 37 °C); right: specific activities of *Cr*HydA1 in TRIS (0.02 M, pH 8) with DT (0.1 M)/MV (0.01 M) and [**APBPy**]$^{2+}$ (5 mM), respectively (400 ng/mL enzyme, 5 min, 37 °C).

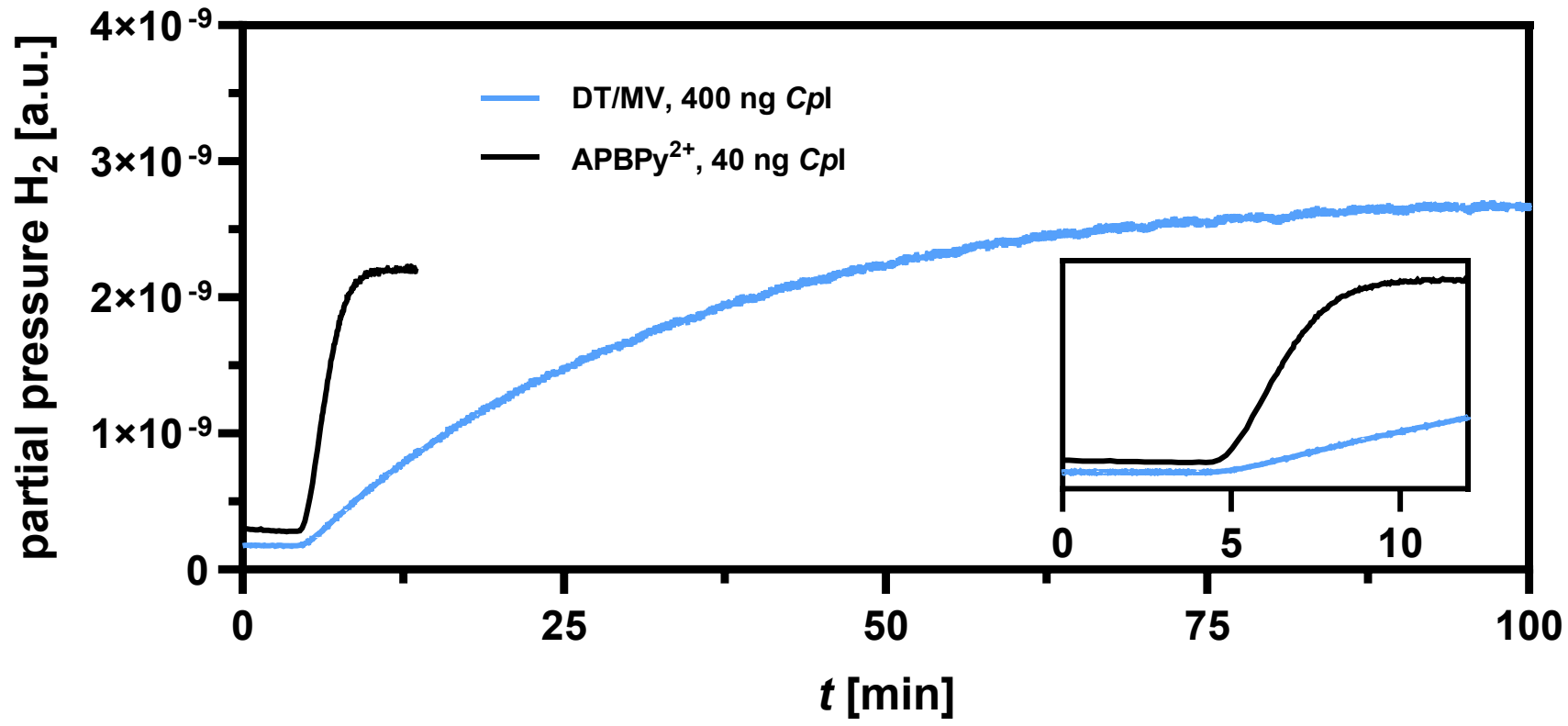

Figure S17: Partial pressure of H_2 *vs.* time from real-time MS measurements of *Cp*I activity assays. Blue trace: 400 ng/mL enzyme in TRIS (0.02 M, pH 8) with DT (0.1 M) and MV (0.01 M) as electron donor/mediator (400 ng/mL enzyme, 37 °C). Black trace: [**APBPy**]$^{2+}$ (5 mM) as electron donor in TRIS (0.02 M, pH 8, 40 ng/mL enzyme, room temperature). Inlet: Zoom into the first 12 min of both experiments, to see initial rates.

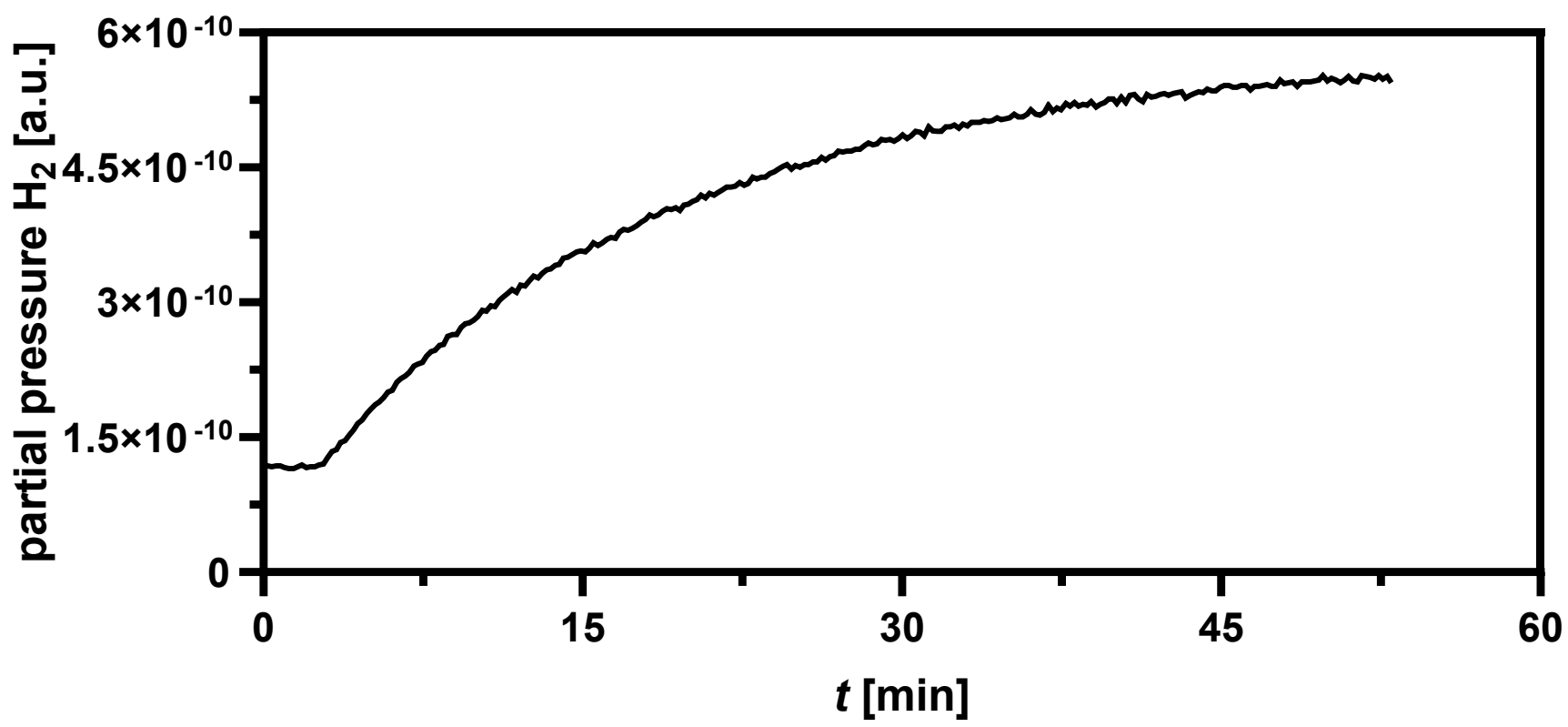

Figure S18: Partial pressure of H_2 *vs.* time from a real-time MS measurement of *Cp*I with [**APBPy**]$^{2+}$ (5 mM) as electron donor in TRIS (0.02 M, pH 8, 10 ng/mL enzyme, room temperature).

Table S1: Activities at the given reaction conditions studied throughout this study. [α]electrochemically reduced to $MV^{+\bullet}$.

Exp.	Enzyme	DT [mM]	$c_{MV^{2+}}$ [mM]	$c_{APBPy^{2+}}$ [mM]	t [min]	T [°C]	m_{enzyme}/V [ng/mL]	Specific activity [μmol_{H_2} $mg_{enzyme}{}^{-1}$ min^{-1}]	Fig.	Buffer	pH
TCD	*Cp*I	100	10	-	20	37	400	1968	S16	MOPS	7
TCD	*Cr*HydA1	100	10	-	20	37	400	330	S16	MOPS	7
n.d.	*Cp*I	-	-	5	n.d.	25	400	n.d.	text	TRIS	8
TCD	*Cp*I	10	1	-	5	37	10	1116	5	TRIS	8
TCD	*Cp*I	-	-	5	5	37	10	10480	5	TRIS	8
TCD	*Cp*I	-	10^{α}	-	5	37	10	650	5	TRIS	8
TCD	*Cr*HydA1	100	10	-	5	37	400	85	S16	TRIS	8
TCD	*Cr*HydA1	-	-	5	5	37	400	47	S16	TRIS	8
RTMS	*Cp*I	100	10	-	-	37	400	-	S17	TRIS	8
RTMS	*Cp*I	-	-	5	-	25	40	-	S17	TRIS	8
RTMS	*Cp*I	-	-	5	-	25	10	-	S18	TRIS	8

References

(1) Sanchez, M. L. K.; Sommer, C.; Reijerse, E.; Birrell, J. A.; Lubitz, W.; Dyer, R. B. Investigating the Kinetic Competency of CrHydA1 [FeFe] Hydrogenase Intermediate States via Time-Resolved Infrared Spectroscopy. *J. Am. Chem. Soc.* **2019**, *141*, 16064–16070.

(2) Khushvakov, J.; Nussbaum, R.; Cadoux, C.; Duan, J.; Stripp, S. T.; Milton, R. D. Following Electroenzymatic Hydrogen Production by Rotating Ring-Disk Electrochemistry and Mass Spectrometry. *Angew. Chem. Int. Ed. Engl.* **2021**, *60*, 10001–10006.

(3) Kuchenreuther, J. M.; Grady-Smith, C. S.; Bingham, A. S.; George, S. J.; Cramer, S. P.; Swartz, J. R. High-yield expression of heterologous [FeFe] hydrogenases in Escherichia coli. *PLoS One* **2010**, *5*, e15491.

(4) Webb, S.; Veliju, A.; Maroni, P.; Apfel, U. P.; Happe, T.; Milton, R. D. Mesoporous Electrodes Enhance the Electrocatalytic Performance of [FeFe]-Hydrogenase. *Angew. Chem. Int. Ed. Engl.* **2025**, *64*, e202416658.
